# Ensuring vaccine potency and availability: how evidence shaped Gavi's Immunization Supply Chain Strategy

**DOI:** 10.1186/s12913-022-08616-9

**Published:** 2022-10-07

**Authors:** Wendy Prosser, Karan Sagar, Michelle Seidel, Soumya Alva

**Affiliations:** 1 John Snow, Inc, Washington, DC USA; 2 Gavi, the Vaccine Alliance, Geneva, Switzerland; 3 UNICEF, New York, USA

**Keywords:** Immunization, Supply chain, Strategy, Gavi

## Abstract

**Background:**

In 2014, Gavi and partners developed a global Immunization Supply Chain (iSC) Strategy, 2015–2020, which prioritized functioning cold chain equipment (CCE) and additional storage capacity. In 2016, Gavi launched the Cold Chain Equipment Optimization Platform (CCEOP) as a funding mechanism to improve CCE availability. In 2018, Gavi commissioned an evaluation of CCEOP in Guinea, Kenya and Pakistan. The global iSC Strategy has recently been revised, drawing on findings from effective vaccine management assessments and practical experiences. This case study presents the CCEOP evaluation and how its findings reinforced the revision of the iSC strategy.

**Methods:**

The CCEOP evaluation used a prospective mixed-methods research design in all three countries involving key informant interviews at multiple levels of the health system, document reviews, direct observation (as and when possible), and a health facility assessment.

**Results:**

Results show that CCEOP was effective at increasing the number of available and reliable CCE, and establishing improved management processes using the project management team (PMT) approach for country management systems and the service bundle provider approach for installation and maintenance. CCEOP also extended the iSC and immunization services in countries. The evaluation results also show gaps in the overall supply chain system, including CCE maintenance.

**Discussion:**

Gavi has recently revised its iSC strategy, which has addressed gaps identified through assessments and practical experiences from stakeholders. Results of the CCEOP evaluation reinforce many of these findings. The strategy now provides more emphasis on supporting the fundamental infrastructure and establishing strong processes for maintenance. It also emphasizes strategic planning and forward thinking for iSC decisions, building on the processes established for the PMT through CCEOP. The original iSC strategy was an impetus for the establishment of CCEOP. The new strategy reflects shifting trends and priorities to fill gaps identified through practical experience, advocated for by stakeholders and thought leaders engaged in the iSC, and validated by the evaluation. It demonstrates the importance of aligning stakeholders with clear objectives and a sound strategy.

**Supplementary Information:**

The online version contains supplementary material available at 10.1186/s12913-022-08616-9.

## Background

Since the launch of the Expanded Program on Immunization (EPI) in 1974, vaccinations against preventable diseases have saved millions of lives; reduced the burden of disease; and contributed significantly to improving the health and well-being of people around the world [1–3]. As a global health priority, investments have been shaped by strategic frameworks, one of which was the Global Vaccine Action Plan for 2011–2020. Hundreds of immunization stakeholders contributed to this plan, which aims to strengthen routine immunization, introduce new and improved vaccines, and advance research and development for new vaccines and technologies [4]. At the same time, the Global Vaccine Action Plan was bolstered by similar goals of Gavi, the Vaccine Alliance, and its third five-year strategy (2011–2015), committed to the uptake and use of underused and new vaccines, strengthening the health system and financial sustainability for immunization, and shaping vaccine markets [5].

These strategies recognized the importance of the immunization supply chain (iSC) to ensure the availability of potent vaccines, yet also acknowledged that many of the supply chains were falling short of expectations and performance. The iSC encompasses all the people, activities, resources, infrastructure, and planning necessary to ensure safe and effective vaccines reach those who need them. During the same time period, the results of the Effective Vaccine Management (EVM) assessment of 2009–2014 across 82 countries indicate that there was a broad range of performance across the nine criterion and all levels of the supply chain; yet, with the exception of the national level for storage capacity, none of the criterion had a median score above the target 80% [6].

**Fig. 1:**
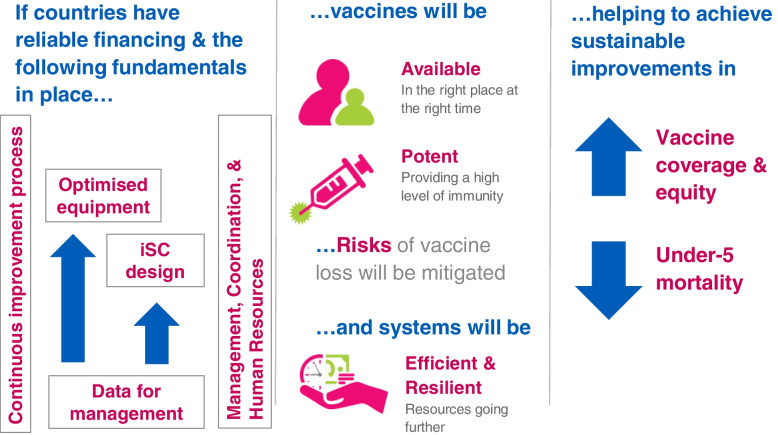
Immunization Supply Chain Strategy and Theory of Change, 2015–2020

In response to this gap in performance of the iSC and the recognition that investments in vaccines must be accompanied by investments in supply systems to ensure availability of potent vaccines, Gavi and its partners developed an iSC Strategy (2015–2020) (Fig. 1) framed by five fundamentals: leadership, continuous improvement, data for management, cold chain equipment, and supply chain system design [7]. The Gavi board approved this strategy in 2014. The strategy highlighted cold chain equipment (CCE) due to its requirement for vaccines and the need for its reliability across Gavi-supported countries.

In June 2015, the Gavi board approved the creation of the Cold Chain Equipment Optimization Platform (CCEOP) as a catalytic investment to help countries modernize and extend the cold chain with reliable and high-performing equipment at an accelerated pace. The CCEOP introduced approaches such as establishing project management teams (PMT) within the ministries of health (MOH) to plan and provide oversight of equipment installation, and using private sector service bundle providers (SBPs) to deploy and install the equipment, including voltage stabilizers. These investments were to contribute to improved immunization coverage and equity and catalyze the development of optimal and better performing technology to meet countries’ needs.

Since its introduction and through the end of 2020, Gavi has invested more than $239 million in CCEOP to commission more than 53,000 pieces of CCE in more than 50 countries, using UNICEF for project management and as the procurement agency. In 2018, Gavi commissioned an evaluation of the CCEOP to assess the progress of CCEOP against its original objectives and to understand details of the deployment process. The evaluation, completed in mid-2021, will inform future CCE deployments globally through CCEOP. As the new equipment has been deployed, advances have been made in other supply chain fundamentals, and global contexts have shifted (e.g., in response to the COVID-19 pandemic).

In June 2020, the Immunization Supply Chain Steering Committee, co-chaired by UNICEF and Gavi, began to revise the iSC strategy to reflect these changes and update priorities accordingly. The development of the iSC Strategy (2021–2025) was completed in March 2021. Stakeholders used evidence from the EVM assessment scores from 2009–2020, with 44 countries represented in 2020, to identify iSC areas that improved during the implementation of the 2015–2020 strategy and those that were lagging [8]. Stakeholders also took guidance from the Immunization Agenda 2030, developed by the World Health Assembly with the support of countries and partners to establish a new global vision and strategy to ensure everyone has access to life-saving vaccines [9]. At the same time, the Gavi board endorsed its Phase 5 Strategy (2021–2025), also reflecting the imperative to ensure no one is left behind with immunization [10]. The findings and priorities from these analyses, as well as practical experience from key stakeholders involved in the iSC, both at global and country levels, guided the development of the iSC strategy. The results of this CCEOP evaluation validated many of the findings and priority settings for the strategy.

The main objective of this paper is to track how practical experience from implementation of the original iSC strategy, global shifts, and lessons learned have shaped the development of the new strategy, reinforced by the findings of the CCEOP evaluation, to reflect the current context and priorities for immunization programs and supply chains. This paper contributes to existing literature by validating progress to date and priorities as identified in the new iSC strategy.

## Methodology

The CCEOP prospective evaluation using a mixed methods study design in Guinea, Kenya, and Pakistan assessed the effect of CCEOP on CCE and the iSC, based on five evaluation themes: relevance, effectiveness, efficiency, outcomes/results, and sustainability.

The country-level evaluation consisted of four time points for data collection in each country: a baseline (May–July 2018); two midline (November 2018–March 2019, and September–December 2019); and an endline (December 2020–February 2021). The MOH in each country approved the evaluation and internal institutional review boards found the evaluation exempt from human subject oversight since it involved survey activities without identifiers or sensitive questions.

The baseline assessment focused on gauging the situation prior to deployment of CCEOP equipment and evaluating the planning process. The midline captured changes through the post-deployment period, including the effect on selected outputs. The endline evaluation focused more on understanding the situation across the three countries soon after CCE was installed under the CCEOP; and comparing differences between baseline and endline. The endline examined the effects and expected outcomes of CCEOP, along with a focus on overall systems strengthening.

The evaluation also considered market-shaping activities, but this component is out of scope for this manuscript.

The methodology to develop the iSC strategy involved global- and country-level stakeholders reviewing the scores of EVM assessment, strategic guidance, and practical experience. The results of this evaluation reinforced the findings and insight from the strategy development process.

### Data sources

The evaluation obtained data from a variety of sources including document review, direct observation of the CCEOP planning and implementation process (when possible), key informant interviews (KIIs), and a health facility assessment (HFA). Documents reviewed in each country included CCEOP applications, operational deployment plans, CCE inventory and gap analysis, PMT meeting notes, reports on deployment, EVM assessment reports, comprehensive multi-year plans, immunization program reviews, and correspondence between Gavi and MOH. Data from the health management information systems/logistics information management systems could not be used to the extent intended because of problems with quality and access at the sub-national level.

The qualitative component included KIIs at different levels of the health system, from the national to the health facility, and the SBPs responsible for CCE installation and maintenance during warranty in each country. The KIIs were conducted using semi-structured interview guides customized for respondents at the various levels of the health system. Different questions were asked in qualitative interviews at each data collection point in time of this prospective evaluation to address relevant questions at each timepoint.

The quantitative component was an HFA in selected facilities in the sampled districts. The purpose of the HFA was to establish a follow-up measure of indicators at facilities and district stores. At endline the HFA also included 60-day temperature data downloaded from 30-day temperature recorder devices using the Varo application to assess the CCE ability to maintain the appropriate temperature, using 2–8° C as the ideal range for vaccines at the facility level.

CCE inventory was collected at baseline and endline. Freezers and freeze space was not included in this assessment. Non-PQS approved CCE was included as a category of equipment only; capacity utilization rate was not calculated for this type of equipment because it is not recommended for vaccines.

### Study area and sampling

The purposive sampling approach was somewhat consistent across the three countries to facilitate cross-country comparison. Differences in the nature and timing of CCE deployment through CCEOP resulted in variations in the sample design in each country. Furthermore, because it was not feasible to conduct the evaluation in all areas receiving CCEOP support, the approach focused on obtaining in-depth information from selected regions in each country. The final sampling areas at the district/sub-county and health facility levels for baseline and subsequent data collection points in time were selected using criteria described below, varying slightly by country. In general, this yielded a mix of high and low CCEOP coverage districts/sub-counties in each of the selected provinces/regions/counties across the three countries.

The small sample size of health facilities based on their receiving CCEOP equipment is an acknowledged limitation.

In Guinea, three regions were selected: Boké and Faranah, with the highest projected number of facilities receiving equipment through CCEOP; and Kankan, with the second-lowest CCEOP coverage. The endline sample included 110 health facilities, health centers (HCs), and health posts (HPs), and 12 district depots by region for the HFA. Health posts were prioritized for CCEOP deployment. At endline, 69 KIIs were conducted with stakeholders at each level of the system (Table 1).

**Table 1:** KIIs implemented in each country at endline data collection

**System Level**	**Interviewees in Each Country**	**Guinea**	**Kenya**	**Pakistan**	**TOTAL**
National	EPI senior management, PMT, partners, SBP; finance management specialist (Pakistan)	11	7	7	**25**
Province/ County/ Regional	EPI program managers, logisticians, cold chain technicians, health directors; depot store managers (Pakistan); EVM coordinators (Pakistan)	8	9	6	**23**
District/ Sub-county	Medical officer, cold chain technician, EPI focal person; health director (Guinea)	17	27	24	**68**
Health facility	Facility in-charge; vaccinators (Pakistan); community health workers (Pakistan)	36	20	48	**104**
**TOTAL**		**72**	**63**	**85**	**220**

In Kenya, Marsabit, Homa Bay, and Kitui counties were included based on criteria that included remoteness, high number of facilities receiving equipment through CCEOP, priority status for equipment rollout designated by the MOH; full vaccination coverage below the national average of 75 percent (Kenya National Bureau of Statistics and ICF International 2015); and various safety and security considerations. The final sample for the endline HFA included 136 health facilities and 13 sub-county stores, for a total of 149 facilities. At endline, 63 KIIs were conducted with immunization program staff, SBPs, and partners at each level of the system.

In Pakistan, the evaluation was conducted in Sindh and Punjab Provinces where CCEOP equipment distribution was high in general, from which six districts were selected based on immunization coverage rates for pentavalent and measles-containing vaccine (MCV) from the Multiple Indicator Cluster 2014 district-level data to categorize (high, medium, low coverage) to ensure each category was represented in the sample. The sample size and selection of facilities for the HFA were determined at baseline. All facilities that were part of the baseline and midline assessments were evaluated at endline. The sample was stratified by facilities receiving CCE earlier versus later and then a selection of facilities was made. This process resulted in 72 facilities selected for each province and 144 facilities in total planned for the sample. However, a few facilities could not be reached in Sindh, resulting in a sample of 140. At endline, the research team conducted 85 KIIs with stakeholders at each level of the system.

### Considerations for data analysis plan

Data from the evaluation were analyzed to answer relevant questions at each stage related to CCEOP implementation in each country and its effects. The analysis also documented aspects of the CCEOP planning and implementation process, including deployment and details about maintenance, repairs, and warranty.

Several evaluation parameters were considered. First was availability of CCE and capacity utilization, comparing baseline to endline. Capacity utilization was calculated using the vaccine quantity required based on the EPI schedule (e.g., vaccines and number of doses required of each); stated distribution schedule (e.g., monthly distribution to facility level); target population of each facility; and vaccine characteristics (vial size, cubic liters per dose, wastage rate, buffer stock). This was assessed against the total net cubic liters of the performance, quality, and safety (PQS)-approved CCE available and functioning at the facility and used for vaccines. Utilization categories were defined as appropriate if 10–80% of capacity was used; underutilized if < 10% of capacity was used; and as constrained if > 80% of capacity was used. Appropriate capacity use is considered ideal. Next was the frequency of immunization services providing any vaccines on a given day as reported by health workers. The stock availability parameter considered stockouts using tracer vaccines of pentavalent or MCV as reported at the end of the month. Additional parameters included the effectiveness and quality of the SBPs, the overall maintenance system, functioning of the CCE, the strength of the long-term maintenance system, and finally, sustainability through country ownership and equipment reliability.

## Results

The Gavi board approved of CCEOP based on five expectations of the platform [11]. The findings of the evaluation reflect progress as well as gaps in meeting those expectations.

### Expectation 1: safeguard the potency of vaccine stock

Within this expectation, the evaluation considered the number and type of equipment deployed in each of the three focus countries and analyzed the CCE capacity to serve the target population with the current vaccine schedule. Results show that this expectation was largely met.

Results show that CCEOP substantially increased availability and capacity of the cold chain system in the three countries. According to the operational deployment plan for each country, CCEOP has deployed and installed 9,281 ice-lined refrigerators and 3,875 solar direct drive equipment, with expected deployment of 1,264 ice-lined refrigerators and 425 solar direct drives later in 2021.

**Fig. 2:**
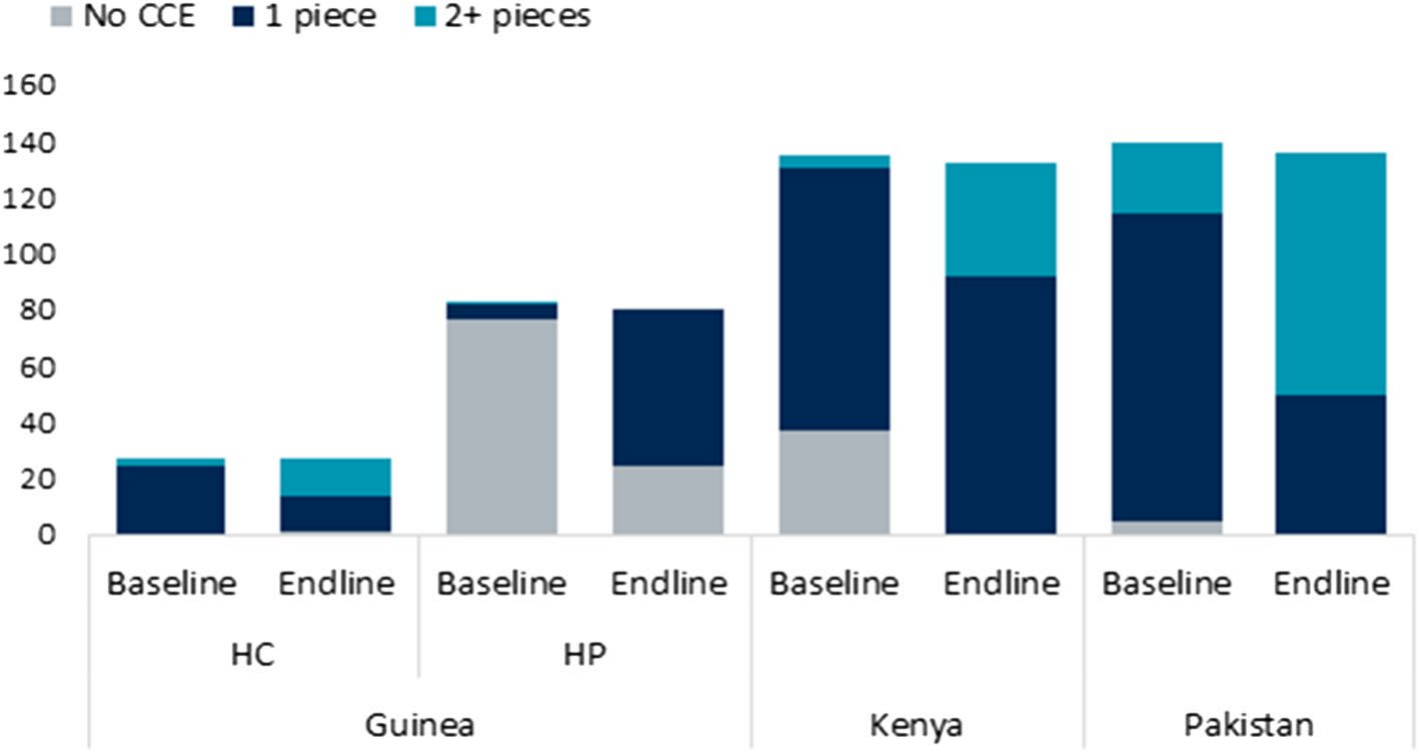
Number of Pieces of CCE in Each Facility, by Study Arm and Time Point

Data from the endline HFA also demonstrated the increase in availability of CCE over baseline (Fig. 2). Particularly notable in Guinea, at baseline, none of the HPs had CCE on premises, compared to the HCs. At endline, the availability of CCE in HPs had increased significantly. In Kenya and Pakistan, the number of health facilities with two or more pieces also increased, providing more CCE capacity to serve their target populations.

**Fig. 3:**
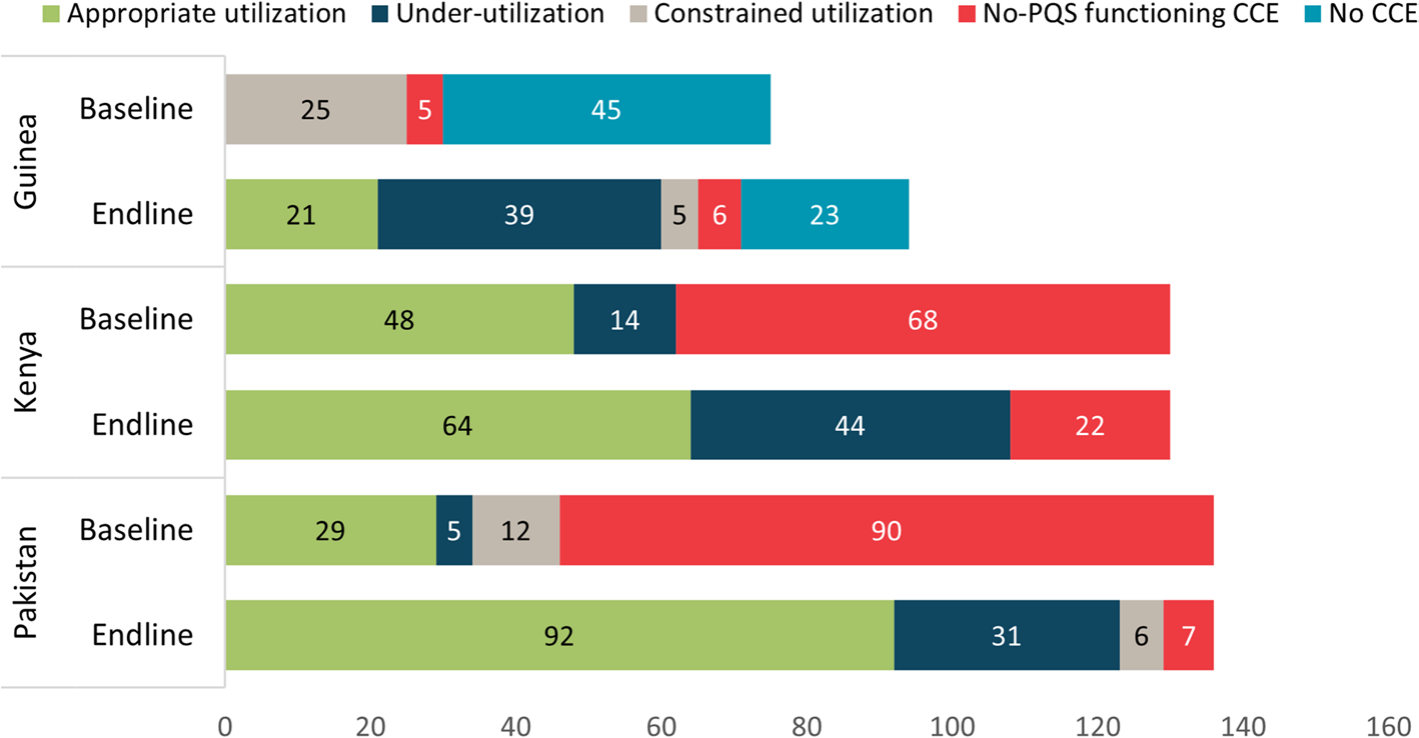
Number of Facilities by Capacity Utilization Category, by Country and Time Point. Utilization category definitions: under-utilization (< 10 percent of capacity); appropriate utilization (10–80 percent of capacity); constrained utilization (> 80 percent of capacity). Appropriate utilization is the most desirable category

The new CCE has resulted in all three countries better using CCE capacity in the appropriate utilization range, with fewer CCE with constrained capacity (Fig. 3). Constrained space indicates minimal flexibility to adjust for disruptions in the supply chain, potentially resulting in stockouts or multiple distributions in a short time frame, leading to inefficiencies. The majority of the equipment is in the “appropriate utilization” category, implying that the CCE is occupying 10–80 percent of its space based on the regular distribution schedule and target population. The “under-utilization” category implies that the current EPI schedule occupies less than 10 percent of the CCE space; however, this allows for growth in the population and number of vaccines (and potentially other cold chain products) provided through the health system. The new equipment has also replaced domestic and non-PQS approved equipment per WHO standards, which is a notable success in ensuring the functionality of the equipment and helping to ensure vaccine potency.

### Expectation 2: prioritize investments that contribute to improved coverage and equity, such as replacing non-functioning equipment and extending the reach of immunization services

**Fig. 4:**
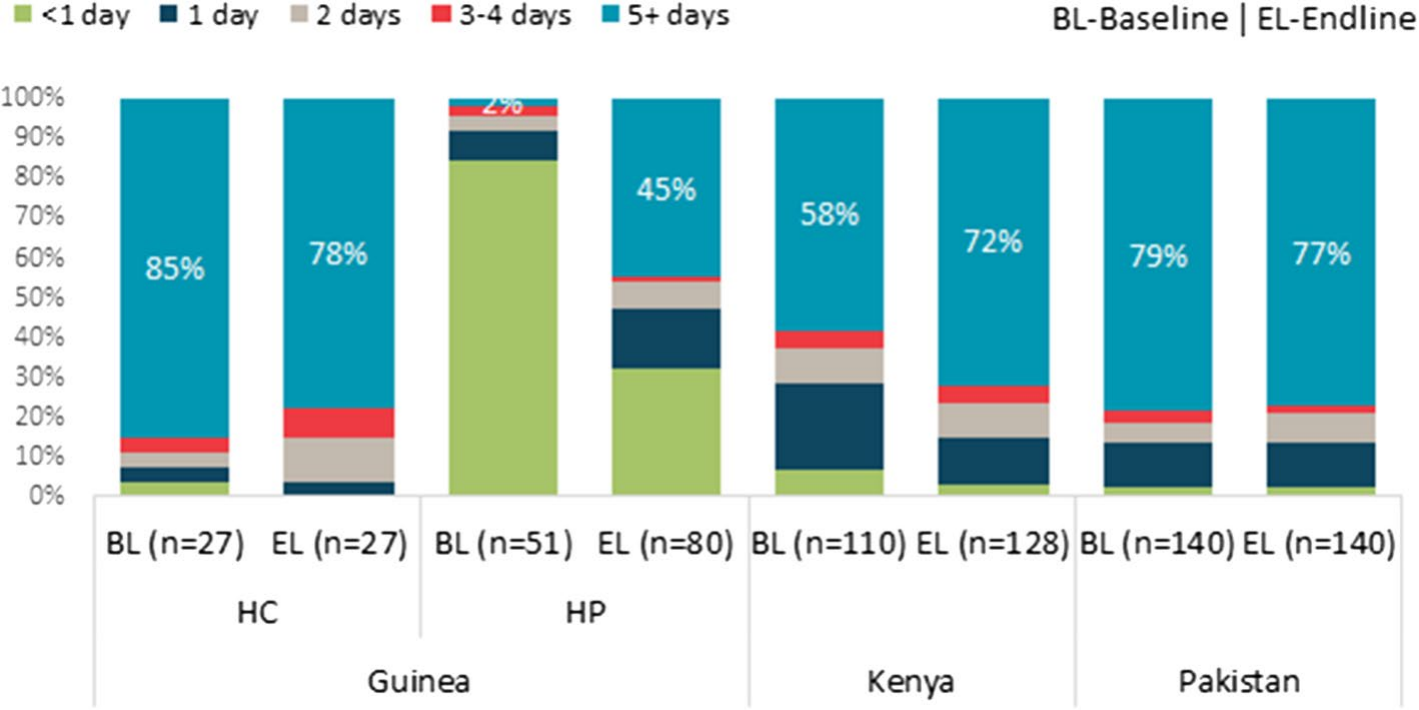
Frequency of Immunization Services in Health Facilities, by Country and Time Point

HFA data from the sampled facilities at baseline and endline showed that the frequency of immunization services of any vaccine offered in health facilities remained consistently high or increased over time in sampled program facilities that received CCE in all three countries (Fig. 4). At endline, 76 percent of facilities in the program group in Kenya offered immunization services five or more days a week, compared to 53 percent of facilities at baseline. Improvements in Guinea, though lower, were evident, especially in HPs that were the focus of the initial deployment of CCEOP because most did not have CCE prior to CCEOP and were collecting vaccines using small carriers on special immunization days.

**Fig. 5:**
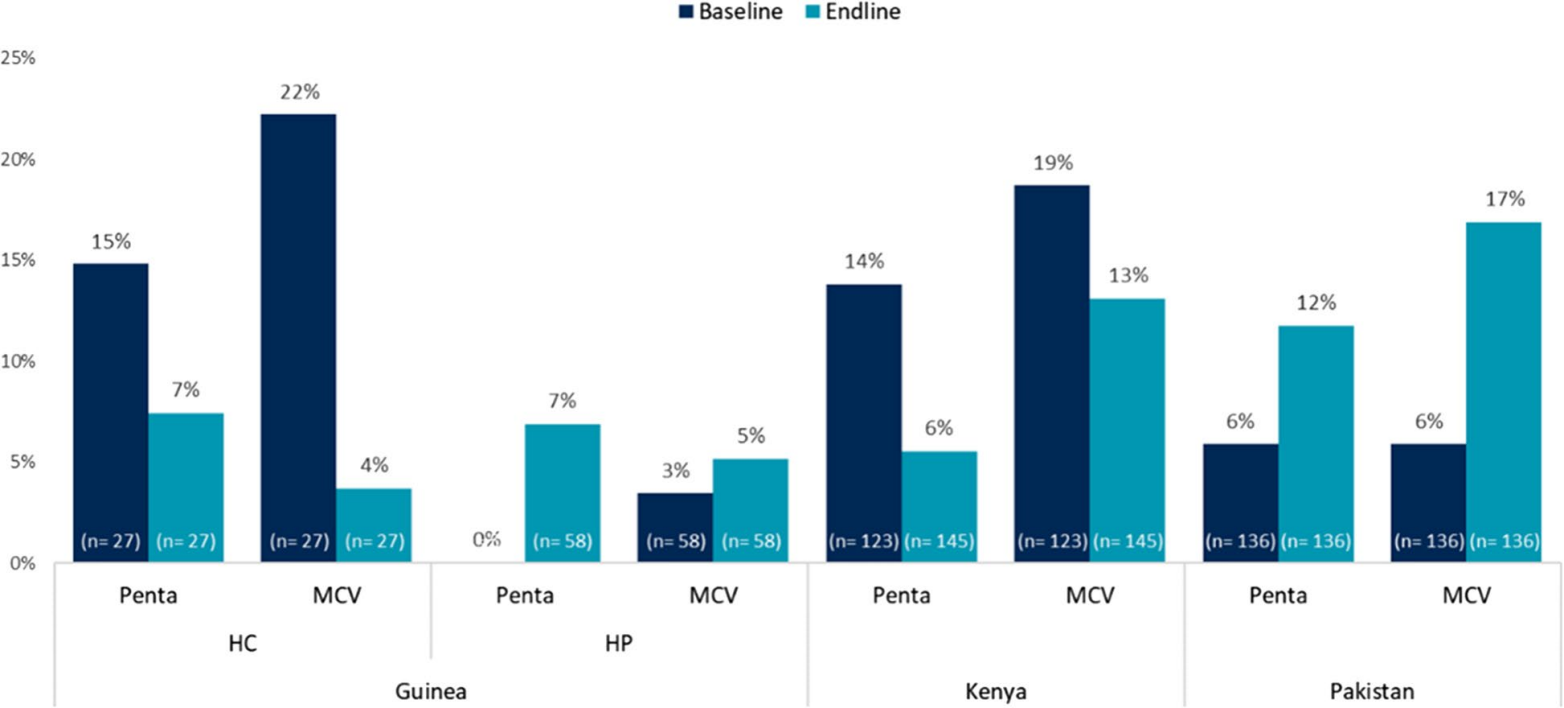
Percent of Facilities Reporting Stockout of Pentavalent and MCV, by Study Arm and Time Point

The HFA provided information on stock availability of pentavalent and MCV as tracer vaccines, and results indicated that stockouts continued throughout the evaluation, regardless of the availability or capacity of the CCE (Fig. 5). Results were inconsistent across the countries. National-level stockouts of a few vaccines occurred during the evaluation timeline, caused by either delayed payments or disruptions in the global supply chain due to the COVID-19 pandemic. At the facility level, fewer stockouts of pentavalent were seen at midline, yet more stockouts of MCV were seen at the same time. Stockouts decreased in general in facilities in Kenya and Guinea over the course of the evaluation, although exceptions were noted. A similar decline was not evident in Pakistan. Reasons for this are unclear, but it reaffirms that there are many other influencers of stock availability, including national-level stockouts and distribution systems, capacity of staff to manage stock and submit orders, and the need to update forecasted demands with the extended reach of immunization services.

### Expectation 3: shape the CCE market to catalyze the development of optimal equipment and introduce the service bundle approach

CCEOP established the service bundle approach as an innovative mechanism whereby CCE manufacturers are accountable for contracting local SBPs for delivery, installation, and maintenance of equipment under warranty, and training local technicians on maintenance. The evaluation sought to determine the effectiveness of the SBPs on installation, reporting requirements, responding to warranty issues, and building capacity of government cold chain technicians.

Midline results from the KIIs showed that respondents were mostly satisfied with the quality of services provided by the SBPs for installation of the CCE, as it streamlined the delivery and installation process and removed the burden on the national government. The SBPs followed operational deployment plans developed by each MOH that identified the facility, location, and type of CCE to install. While each country experienced a few deviations from the ODPs, the SBPs were quite flexible, responsive and able to adjust to changes with minimum disruptions in a timely manner, and without reported additional costs.

An additional finding from midline was the effectiveness of the robust system of monitoring and documentation required for the SBPs. Across the three countries and those who participated in the KIIs, this monitoring and documentation system was effective for tracking equipment and ensuring accountability from the SBPs. This allowed the PMT and UNICEF to monitor activities and receive regular updates.

Kenyan stakeholders expressed concern over the additional cost of the SBPs, noting that money could have been better spent procuring additional equipment at lower costs, and argued that they have sufficient internal capacity to do the same work as the SBPs. This was also noted by stakeholders in Guinea but was not a concern in Pakistan.

Despite the successful approach for installation, endline results showed inconsistencies across the SBPs for providing ongoing maintenance, as determined by the terms and conditions of the warranty. In Kenya and Pakistan, stakeholders reported less satisfaction for the quality of SBP services for on-going maintenance and support. Conversely, one SBP in Guinea demonstrated exceptional services, providing routine preventive maintenance to the facility level and responding to corrective maintenance needs.

Stakeholders noted lack of clarity of CCE manufacturer warranties and post-installation role of the SBP. Respondents understood that the warranty does not cover anything caused by negligence of an operator or that has been altered by another (unauthorized) technician; but beyond that, there was widespread uncertainty on what was covered.

The SBPs were tasked with training government technicians to provide CCE maintenance after the warranty ended. Respondents expressed appreciation for SBP assistance with training government technicians and ensuring that, in many cases, they were included in maintenance visits to reinforce training and skills. Despite the appreciation, the need for additional training, including on-the-job, was a recurring need across the three countries and at each level of the health system. Health facility staff reported insufficient training from the SBPs for preventive maintenance. Additionally, cold chain technicians at higher levels of the system in each country expressed interest in more training on corrective maintenance for technical interventions that a piece of equipment may require.

### Expectation 4: incentivize countries to anticipate maintenance requirements in a systematic way and ensure they are met

**Fig. 6:**
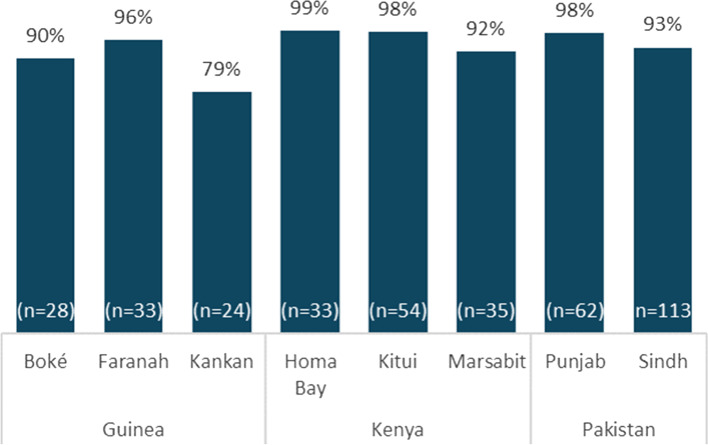
Average Percent of Time CCE Spent in Safe Time at Endline, by Country

CCEOP procured optimal CCE with the expectation of minimum maintenance, which bore out through the evaluation. Endline assessment results show that the majority of the new equipment is functioning well, with the large majority of the CCE maintaining the ideal temperature range, as seen by data collected at endline reflecting the prior 60 days (Fig. 6). This reliable CCE has generally performed very well with little need for corrective maintenance. An exception in the case of a manufacturing error was resolved shortly after installation across affected countries.

While training was provided to health workers and CCE technicians, the overall maintenance system was not strengthened. Through the KIIs, country-level stakeholders expressed concern for long-term maintenance requirements when warranties expire (after two to 10 years, depending on the manufacturer and contract). This concern extends to all CCE in the system, a challenge compounded by multiple CCE models in use across these countries. Having a parallel maintenance system for the CCEOP-procured equipment under warranty has complicated an already weak and under-funded system with unclear processes. In each country, the processes for reporting maintenance issues are unclear and may differ for CCEOP and non-CCEOP equipment. Stakeholders reported challenges with the maintenance system included lack of operational funds and spare parts.

Moreover, having multiple models in the system adds a layer of complexity to ensure that CCE function effectively in the long term. The two or three new models for each country procured through CCEOP are part of the larger CCE system that already had multiple brands and models. Through initial CCE selection during application, each country made an effort to standardize models to reduce the multiplicity of brands, with the goal of rationally managing resources for maintenance technician capacity development and spare parts. According to the CCE inventory submitted at the time of CCEOP application for each country, Kenya had 20 different models in use across the different levels of the health system, Guinea had 22, and Pakistan had 55. Domestic models not approved by WHO were also in use for vaccine storage to some degree in each country. One concern raised by stakeholders was that the multiple models in use require different spare parts and technical expertise to maintain, complicating an already poorly performing maintenance system.

Decommissioning of old equipment was also raised as a concern. A decommissioning plan can identify safe ways to dispose of equipment that is no longer functioning, or repurpose the parts for other CCE. While stakeholders recognized the importance of developing a decommissioning plan, none of the countries had completed one at the time of data collection.

### Expectation 5: contribute to ensuring the sustainability of programs by supporting countries to use more reliable and efficient equipment, which has over-all lower recurrent and lifetime ownership costs

Results related to sustainability—and by extension system strengthening—are mixed. In terms of CCE that is reliable, efficient, and requires less maintenance in the immediate future, CCEOP has been quite successful. This has introduced efficiencies to the system, particularly notable in Guinea where CCE was placed in facilities that did not have equipment. The new equipment has reduced the burden on health workers to periodically collect vaccines for special immunization days. It has also brought the vaccines closer to the community, more reliably and regularly, thus reducing the distance that people in some communities must travel for vaccines at a far-away health facility.

In terms of leadership and country ownership as an indication of sustainability, the establishment of the PMT created a decision-making system for CCEOP planning and implementation monitoring. Closely aligned with the National Logistics Working Group, the PMT in each country was able to prepare the application, develop and revise the operational deployment plan, and work closely with the SBPs for installation and monitoring. This decision-making structure of the PMT, however, did not cascade to the sub-national levels in two countries (Kenya and Guinea), leaving gaps in ownership, involvement, and coordination at the lower levels. In Guinea, while the PMT was closely involved in the application, preparation, and deployment, it was less active at endline, with attention shifted to responding to the COVID-19 pandemic. This was true for the National Logistics Working Group as well.

The results of the evaluation also highlighted gaps in the overall health system that, while not the focus of CCEOP, indicate the need for a systems approach to strengthening and sustainability. The health system did not keep pace with the need for more resources as the CCE was deployed and immunization services expanded. Upon deployment and since resolved, Guinea and Kenya experienced a few situations where facilities received new CCEOP equipment but had no staff trained to administer vaccines.

Interestingly, in Guinea, the significant investment in the cold chain has expanded the supply chain and increased focus on logistics, communication, coordination, and other aspects of the immunization program. Further support is being provided to strengthen performance of the national program by restructuring EPI, including recruiting additional staff. The increase in CCE availability has highlighted the need for staff able to handle vaccines and CCE, and provide immunization services.

## Discussion

CCEOP was designed to respond to one of the five fundamentals of the iSC strategy related to increasing the availability of reliable CCE, and it has done exceptionally well at achieving this goal across health systems in Gavi-supported countries. The results of the CCEOP evaluation reflect improved EVM assessment scores through 2020, particularly in the category of cold chain storage capacity, which now scores above 80 percent [8]. The results of the EVM assessments also highlight areas that are lagging, such as CCE maintenance and distribution systems, both of which are findings of the CCEOP evaluation, which noted gaps in maintenance systems and persistent stockouts potentially linked to the distribution system.

The Gavi board has approved a new overall five-year strategy, 2021–2025 (Gavi 5.0) with a vision to leave no one behind with immunization and an emphasis on reaching zero-dose children, defined as those who have not received any routine vaccine. These global shifts, changing priorities, and achievements of the iSC strategy and performance gaps shaped the development of the iSC strategy for the same period.

**Fig. 7:**
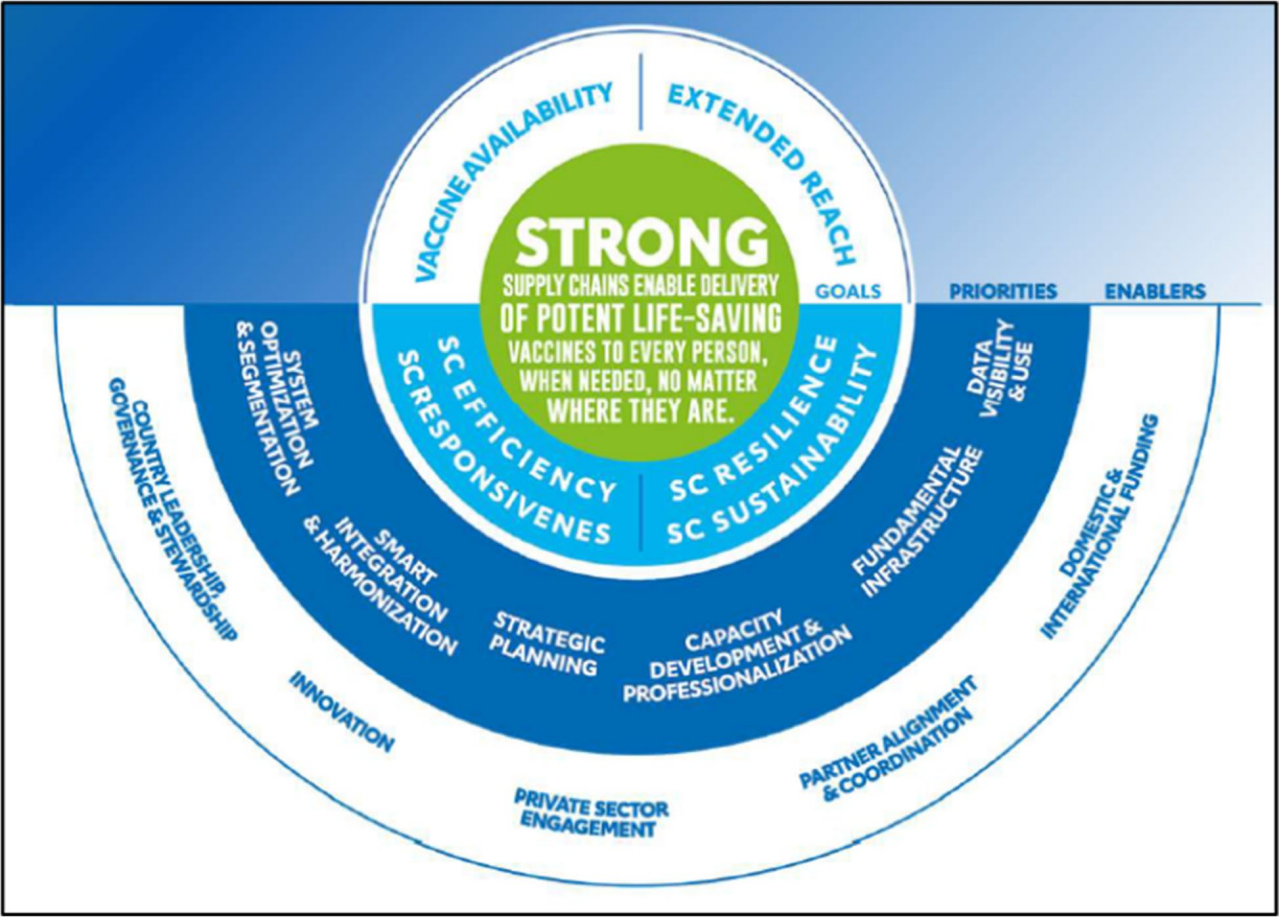
Immunization Supply Chain Theory of Change, 2021–2025

Building on the first iSC strategy (2015–2020), the revised strategy (Fig. 7) recognizes that inconsistent availability of high-quality vaccines and limited reach of iSCs among underserved populations threaten access, immunization coverage, and equity outcomes. The strategy guides action on and advocates for priority needs while promoting global best practices. The strategy seeks to improve alignment with countries, partners, and other donors, encouraging collaboration and complementarity of strengths. It also recognizes the importance of integration with broader supply chain and health systems strengthening efforts, shifting away from siloed immunization efforts. It facilitates investments that are responsive to needs and context and will contribute to stronger supply chain performance. Finally, the strategy strives to ensure accountability by setting and monitoring the quality standard of country performance and technical assistance provided by partners.

An important shift in this new iSC strategy is moving away from five fundamentals of the supply chain that tended to lead to silo strategies and funding. The six investment priorities identified by the updated strategy build on the five fundamentals yet are more holistic, incorporating key supply chain elements required to attain the strategy goals. The strategy strives to boost investments in areas that need the most attention.

The results of EVM assessments, global priorities, and practical experience of stakeholders involved in the iSC around the world were reinforced by the findings from the CCEOP evaluation and shaped the six investment priorities in this way:

1. *System optimization and segmentation* prioritizes the design of the supply chain and individual segments to reach everyone in a cost-effective and efficient manner. It also emphasizes reducing and managing waste. Shifting the priority in this way has sharpened the focus on extending the reach of the supply chain, reflecting Gavi’s overall priority of reaching zero-dose children. This also reflects the advances made by CCEOP and extending the supply chain reach, particularly notable in Guinea, and expanding immunization services, noted across the three countries. It also emphasizes the fact that an iSC is more than just CCE. Linked to managing waste, decommissioning old CCE has been a gap in recent years and is an important recommendation from the CCEOP evaluation; it is salient that it is included in the strategy.
2. *Data visibility and use* reflects the more practical aspects of data management: the need for digital systems to enable data visibility throughout the supply chain. It also emphasizes the processes and tools required to support the use of high-quality data for decision-making to drive continuous supply chain performance and improvement. This reflects the shift to invest in remote temperature monitoring devices for the CCE, providing real-time visibility into equipment performance. This is a notable change from the previous strategy, which simply recognized the importance of having reliable data without a focus on how to collect and manage those data. This priority is driven by the negligibly improved EVM assessment scores of information systems (63 percent in 2015 and 64 percent in 2020) and builds on new technologies that can support data-driven decision making related to improved supply chain management and CCE selection and maintenance.
3. *Capacity development and professionalization* prioritizes building local supply chain talent in partnership with local organizations. While this relates to all aspects of supply chain management and leadership, it is relevant for CCEOP for CCE maintenance, inventory management, gap analysis, and rehabilitation planning. The need for additional training for corrective and preventive maintenance was an important finding from the CCEOP evaluation across all countries. This may reflect many things, including staff turnover and the ineffectiveness of the training methods used during CCEOP implementation. It implies that capacity development becomes institutionalized in countries with the ability to adopt methodologies that leverage new technologies, reinforce learning and expertise in a practical way, and emphasize on-the-job training to complement staff on-boarding. This may also encompass the capacity to manage private sector partnerships, such as the SBP model, and build on their successes while correcting shortcomings.
4. *Fundamental infrastructure* is most relevant to the CCEOP evaluation. Most notably, the priority shifts from a focus on CCE only to prioritizing continued support to maintain CCE capacity and supply chain infrastructure. CCEOP has increased the availability and use of CCE that is functioning well, as demonstrated by the reliable temperature reports. As the evaluation showed and as reflected in the EVM assessment scores, the gap now is in the system to ensure the CCE can continue to function. Maintenance systems need to be revolutionized to protect the investments made to date and address the multiple CCE models in a system [12].
5. *Strategic planning* brings focus to enhanced country ownership and leadership to develop and finalize national multi-year supply chain operational and strategic plans. Plans should define priorities and interventions that incorporate the needs of the people while allocating responsibilities. This element should map progress toward a common vision for the country’s iSC. This priority builds on the success of the CCEOP in setting up the PMT, and establishing procedures and processes for planning and monitoring deployment, including coordinating with SBPs. Guinea provides an excellent model for how supply chain strengthening can then extend to leadership for the overall immunization program.
6. *Smart integration and harmonization*. This new area of focus connects people, products, programs, and functions in context-appropriate ways to improve efficiency and performance. This priority reflects the global trend of leveraging resources across health program areas when feasible and effective [13, 14]. Related to CCE and integrating other cold chain products, the results of the evaluation showed that capacity utilization of the CCE in the three countries was largely sufficient to accommodate growth in the immunization program or with other cold chain products. It is important to note that this priority qualifies integration as “smart” to be context-specific and managed appropriately.

The strategy recognizes important enablers as critical to reaching the goals and vision. Country *governments* are central stewards, providing oversight for the entire supply chain across sectors, demonstrating leadership and governance. *Innovation* can drive new approaches, tools, and processes that contribute to strengthening the iSC. This may be reflected through *private sector engagement* and *partner alignment and coordination* to minimize duplication and leverage expertise. Finally, *domestic and international funding* are key enablers that must consider context-specific funding cycles.

## Conclusion

The first iSC strategy aligned stakeholders and thought leaders with a common vision and goal. It also guided the development of CCEOP as a key funding mechanism, which advanced certain aspects of that strategy. In an effort to continuously learn, improve, and strive for excellence, the new iSC strategy reflects a shift in priorities based on the achievements as well as gaps in efforts to date. It is with this new strategy that Gavi seeks to ensure that strong supply chains enable delivery of potent life-saving vaccines to every person, when needed, no matter where they are.

## Supplementary Information

**Additional file 1.**

**Additional file 2.**

## Data Availability

The datasets used and/or analyzed during the current study are available from the corresponding author on reasonable request.

## Abbreviations

CCE: Cold chain equipment
CCEOP: Cold Chain Equipment Optimization Platform
EVM: Effective vaccine management
EPI: Expanded Program on Immunization
HC: Health center
HFA: Health facility assessment
HP: Health post
iSC: Immunization supply chain
KII: Key information interview
MOH: Ministry of health
MCV: Measles-containing vaccine
PMT: Project management team
PQS: Performance, quality, and safety
SBP: Service bundle provider
