# Supplementary material for: Ensuring vaccine potency and availability: how evidence shaped Gavi's Immunization Supply Chain Strategy
Source: BMC Health Serv Res. 2022 Oct 7;22:1237. doi: 10.1186/s12913-022-08616-9 (PMC9540167; doi:10.1186/s12913-022-08616-9)
Supplement: Supplementary file 1 — Additional file 1. [file 12913_2022_8616_MOESM1_ESM.pdf]

JSI RESEARCH & TRAINING INSTITUTE, INC.

Evaluation of the Cold Chain Equipment Optimization Platform (CCEOP) - Baseline

Key Informant Interview Guide

April 2018

Submitted by the CCEOP Evaluation Team to Gavi, the Vaccine Alliance

JaRco Consulting

Research And Development Solutions

SVI STAT VIEW International

Interview: Country Evaluation - National Level (NVIP/MoH)

Note: Please include all types of equipment (CCE, RTMD, Cold boxes) being procured through CCEOP.

Introduction

1. Can you please describe to me your role in the CCEOP?

Proposal development

We would like to talk about the CCEOP proposal development process.

2. Can you explain the proposal development process? [*Probe for activities and timeline]*

Addressing gaps in CCE in the country proposal

3. Before developing the proposal, how did you assess the cold chain equipment available in the country and identify the gaps?

4. How did the proposal address those gaps?

5. What were some of the barriers to filling all the gaps? *[Probe: financial, political support, organizational and capacity constraints]*

6. How were counties, sub-counties and health facilities chosen to receive CCE? Can you explain the process and assumptions made? *[Probe: What criteria were used for selection? How do these facilities and location sites relate to coverage and equity?]*

7. When developing the proposal, what kinds of feedback and guidance did you receive from Gavi and other partners (including WHO and UNICEF)? How timely was their feedback?

8. How was their feedback addressed? Who was involved in addressing the revisions, if any, to the CCEOP application guidelines?

9. How often did you communicate with Gavi and the partners during the proposal process? What mode of communication was used to communicate with them?

10. Overall, did you feel that you had sufficient support from Gavi and other partners (including WHO and UNICEF) during the proposal development process? Why or why not? [Probe: *What additional support would have helped you to develop the proposal?*]

11. During the proposal development, to what extent did you feel engaged with the whole process? [Probe: *To what extent did you feel that Gavi and the*

Alliance partners took into account your perspectives? / Were there moments when you wanted to be more involved during the application stage?]

12. Which organizations and people, including those at the county and lower levels, were involved during the application stage of the CCEOP? [*Probe: In what way were they involved?]*

13. Which other agencies or individuals should have been involved, but were not?

Cold chain equipment

14. What types of equipment were chosen? What were the main sources of information and considerations guiding the country's CCE choices in the application process? [*Probe: Why were those considerations made? Did any considerations change over time?]*

15. Was cost one of the considerations? Was there a budget amount allowed? *[Probe: Did that impact any decisions?*] To what extent did these sources of information differ from before the CCEOP?

16. Some of the selected equipment has RTMs. Was the choice of the model dependent on the availability of this feature?

17. How did the most recent CCE inventory influence the way you made equipment choices in your CCEOP application?

18. Did Kenya receive its first choice of equipment? [*Probe: If not, what changes in equipment were made? / If so, what factors enabled Kenya to receive its first choice? What were the influences that swayed the choice of equipment?*]

19. Who will be responsible for maintaining the CCE after they are installed?

20. For the equipment with RTMs, what are next steps to make the RTM devices functional?

21. What plans are there to build the capacity of health facility staff to use and maintain the new equipment? [*Probe for training and training manuals]*

22. Is Kenya getting additional CCE from other sources over the next two years? *[Probe: If so, what are these sources? How is that being coordinated?]*

23. Do counties acquire CCE from other sources? *[Probe: If so, where do they acquire them from?]*

Operational Deployment Plan

24. Can you tell us about the development of the Operational Deployment Plan (ODP)? Were any changes made since the first version? *[Probe for information on changes.]*

25. Who was involved in the development of the ODP?

26. Who is responsible for operationalizing the ODP? What challenges are they facing?

27. What is the role of the Project Management Team (PMT)? Who comprises the PMT? How does that team work for the CCEOP? [*Probe for their role in developing the ODP, implementing it and monitoring it]*

Service Bundle Providers

28. With regard to deployment of CCE, could you please describe to me the role of service bundle providers?

29. How were in-country service bundle providers arranged? [*Probe: How and why were those bundle providers chosen? Do you think this was the ideal arrangement for the country? Why or why not?*]

30. What are the advantages of using service bundle providers? What are the disadvantages?

Satisfaction

31. How would you describe the support you have received from UNICEF/Supply Division? Are you satisfied with the support received from them? [*Probe: Please explain why]*

32. Overall, what is your level of satisfaction currently with the equipment you-are scheduled to receive?) Please explain why.

33. What, if anything, would you change about the proposal development and ODP process? [Probe: *What worked well? What could have been done differently? What are some lessons learnt?*]

34. What changes do you expect to see in the performance of the new CCE compared to the CCE it replaced? [Probe: *In terms of functioning or not, need for preventive maintenance, fixing any problems with the CCE, temperature stability, etc.]*

CCEOP alignment with Gavi services

Our final set of questions relate to other Gavi services and activities in the country.

35. What other support (e.g., vaccines, HSS funding, technical assistance) does NVIP receive from Gavi other than CCEOP?

36. How is CCEOP coordinated with other Gavi activities in the country? [*Probe: What process did the country undertake to manage CCEOP with other Gavi support and activities?*]

Expectations on demand for CCE

Next, we would like to discuss some other aspects related to the market and demand for CCE.

37. In your opinion, what is the current demand for optimal and new technology for CCE in this country?

38. What are your long-term expectations over the next 10 years for the demand for technology-appropriate CCE in this country? [*Probe: Why do you think demand will change in that manner? What factors will affect this demand in the long-term?]*

39. Is there anything else you want to discuss?

Thank the respondent for their time. Turn off the recorder.

Interview: Country Evaluation - UNICEF Country & ESARO Office & Other Partners

Introduction

1. Can you please describe to me your role in the CCEOP? What activities were you involved in? How did you collaborate with NVIP in developing this proposal? What was UNICEF's (ESARO and COUNTRY) role in developing the proposal? What about CHAI's role? [*Probe for membership and participation in national level working groups or teams]*

Engagement with Gavi and other Alliance partners (Baseline)

We would like to discuss the engagement of the UNICEF Country/ESARO office/CHAI with Gavi and other Alliance partners.

2. When supporting NVIP in developing the proposal, did you feel that you had sufficient support from Gavi and other partners (including UNICEF/Supply Division)? Why or why not? [Probe: *What kinds of feedback and guidance did you receive from them? How timely was their feedback? What additional information from them would have better helped you to develop the proposal?*]

3. How often did you communicate with Gavi and the Alliance partners? What were the reasons for this communication? What systems have been set in place for this communication?

4. Can you tell us what mechanism has been set up to communicate feedback on equipment performance and satisfaction levels over time?

Addressing Gaps in the proposal (Baseline)

Now, we would like your views on the country proposal process including selection of equipment. [Note: Please include all types of equipment (CCE, RTMD, Cold boxes) being procured through CCEOP.

5. In the country proposals, what were the stated gaps for improved cold chain equipment?

6. How did the proposal address those gaps?

7. What were some of the barriers to filling all the gaps? *[Probe: financial, political support, organizational and capacity constraints]*

8. How did you prioritize counties, sub-counties and health facilities for new CCE? What were the criteria? [*Probe: How did the most recent CCE inventory influence the way you made equipment choices in your CCEOP application?]*

9. Is the country getting additional CCE from other sources over the next two years? If so, from what sources and how is that being coordinated? [*If not mentioned, probe for whether counties acquire CCE on their own]*

Procurement of equipment

10. What considerations were made when selecting or procuring CCE? [*Probe: Why were those considerations made? Did any considerations change over time? Was cost one of the considerations? Was there a budget amount allowed?*]

11. Is the country receiving its first choice of equipment? If yes, what factors enabled the country to receive its first choice? [*Probe: If not, what changes in equipment were made and why?*]

12. What is your level of satisfaction currently with the equipment you are scheduled to receive? Can you give us some reasons for your response? [Probe: *What changes do you see compared to existing CCE?]*

Operational Deployment Plan

13. Can you tell us about the development of the Operational Deployment Plan (ODP)? Were any changes made since the first version? *[Probe for information on changes.]*

14. Who was involved in the development of the ODP?

15. Who is responsible for operationalizing the ODP? What challenges are they facing?

16. What is the role of the Project Management Team (PMT)? Who comprises the PMT? How often do they meet? How does that team work for the CCEOP? [*Probe for their role in developing the ODP, implementing it and monitoring it]*

17. What, if anything, would you change about the proposal development and ODP process? [*Probe*: *What worked well? What could have been done differently? What are some lessons learnt?*]

Service bundle providers

18. With regard to deployment of CCE, could you please describe the role of service bundle providers?

19. How were in-country service bundle providers arranged? [Probe*: How and why were those bundle providers chosen? Do you think this was the ideal arrangement for the country? Why or why not?*]

20. How will installation be coordinated with the service bundle providers and the MOH? *[Probe: What factors affect this coordination?]*

21. What are the advantages of using service bundle providers? The disadvantages?

Expectations on demand for CCE

Next, we would like to discuss some other aspects related to the market and demand for CCE.

22. In your opinion, what is the current demand for optimal and new technology for CCE in this country?

23. What are your long-term expectations over the next 10 years for the demand for technology-appropriate CCE in this country? [*Probe: Why do you think demand will change in that manner? What factors will affect this demand in the long-term?]*

CCEOP alignment with other Gavi services

Our final set of questions relate to other Gavi services and activities in the country.

24. What other support (e.g., vaccines, HSS funding, technical assistance) from Gavi does the country receive?

25. How is CCEOP coordinated with other Gavi activities in the country? [*Probe: What process did the country undertake to manage CCEOP with other Gavi support and activities?*]

26. Is there anything else you want to discuss?

Thank the respondent for their time. Turn off the recorder.

Interview: Country Evaluation - Service Bundle Providers

Note: Please include all types of equipment (CCE, RTMD, Cold boxes) being procured through CCEOP.

Market Shaping Related (Baseline)

1. What are the different types of CCE relevant to vaccines that are in use in the country? How has this changed over time? Is the existing CCE appropriate for the needs of the country? In what way?

2. Have you seen the demand for CCE change over the past six months? [*Probe*: *In what way? Can you share this data with us?*]

3. Now we would like to specifically discuss CCEOP. Could you please describe to me your role within CCEOP?

4. What is your role in the installation and maintenance of CCE for the CCEOP? [Probe: *If there are maintenance issues, who do you follow up with?]*

5. Can you tell us about your responsibilities during the warranty period?

6. How are the contracts arranged for installation of this equipment? Who are you contracting with? [Probe: *If there are issues with the contract along the way, who do you follow up with? Do you have agents or staff at the county level for installation?]*

7. How have the service bundle agreements affected your outlook for this market?

8. Describe the process of coordination with the manufacturer, and MOH for implementation of the CCEOP in the country.

9. How has the CCEOP affected your ability to negotiate pricing agreements? [Probe: *Has it varied by manufacturer? Can you give me an example?]*

10. Do you foresee any unintended consequences of the new equipment coming into the country through CCEOP?

11. What are your long-term expectations for the demand for CCE using new technology and your role in their installation?

Operational Deployment Plan & equipment installation

We would like to discuss the deployment of CCEOP in the country and installation of CCE.

12. Has the distribution plan been completed? How have you coordinated the development of the distribution plan with the MOH? [Probe: *Who were the*

major actors facilitating the coordination? / In your opinion, how is that coordination process turning out?

13. How was the contract arranged for the installation of this equipment? [*Probe: What considerations were made?*]

14. To what extent is the information provided by the MOH sufficient for successfully and efficiently installing the equipment?

15. In the installation of the equipment, do you expect to face any issues/ challenges? How can these be resolved?

Trainings

16. Will MOH technicians be trained on installation and maintenance? [Probe: *Who will conduct the trainings? How long will the training sessions be? What training materials will be used?*

17. How is training being planned for health workers on preventive maintenance? Will they be given manuals and trained to use them?

18. Is there anything else you want to discuss?

Thank the respondent for their time. Turn off the recorder.

Interview: Country Evaluation – County/Province Level

Note: Please include all types of equipment (CCE, RTMD, Cold boxes) being procured through CCEOP.

Introduction and Proposal development (Baseline)

1. Could you please describe to me your role within CCEOP? In case you are not aware, CCEOP is the mechanism through which NVIP/MOH is procuring and distributing cold chain equipment across Kenya with Gavi assistance over the next 2 years.

2. How were health facilities in your county prioritized for new CCE? What was your role in the process of health facility selection in your county? What role, if any, did you have in the selection of equipment?

Addressing CCE gaps in Proposal (Baseline)

3. What were the stated gaps for optimal cold chain equipment in your county that were addressed in the CCEOP application?

4. How did the application address those gaps?

5. What were some of the barriers to filling all the gaps? *[Probe: financial, political support, organizational and capacity constraints]*

6. How were counties, sub-counties and health facilities chosen to receive CCE? Can you explain the process and assumptions made? *[Probe: What criteria were used for selection? How do these facilities and location sites relate to coverage and equity?]*

7. What was the process for CCE procurement in this county in the past?

Existing CCE and Maintenance (Baseline)

We would first like to talk about the existing CCE in your county.

8. In the past 3 months, how often did you hear about CCE problems? Can you tell us about some of these problems? [*Probe: If there were problems, how were those problems resolved?]*

9. How is CCE generally maintained? Is there a maintenance plan?
 [Probe: What is the role of health staff in maintenance? Are there trained technicians? Do they travel to the facilities or does the equipment come to the county level? How are transport and spare parts provided?]

10. What are some of the challenges with maintaining the cold chain equipment?

11. How does the availability of cold chain equipment impact vaccine availability in your county? *[Probe for examples]*

12. What about the impact on immunization coverage? *[Probe for examples]*

13. And the impact on vaccine wastage rate? *[Probe for examples]*

Distribution Plan

14. What information have you received on the distribution of new CCE in your county? Please provide details of the timeline and distribution plan and your involvement in the distribution.

Relevance and maintenance of CCE

15. Describe the timing and process of installation of new CCE in your county? [Probe: Who is responsible for what actions and the resources available for it?]

16. And what about maintenance? [Probe: Who is responsible for what actions and the resources available for it?]

17. [Probe for the role of service bundle providers/distribution agents?]

Supervision and training

18. What plans do you have for monitoring the installation and performance of the newly installed CCE? [*Probe for equipment, stock management practices, immunization service provision].*

19. Who will be trained in operation and maintenance? [Probe: *Who will conduct the trainings? How long will the training sessions be? What training materials will be used?*

20. How is training being planned for health workers on preventive maintenance? [Probe for use of manuals and training to use them]

Satisfaction

21. Overall, how satisfied are you with the choice of new CCE? Can you explain why?

22. What, if anything, would you change about the planning and proposed implementation process? [Probe: *What is working well? What could be done differently?*]

23. Is there anything else you want to discuss?

Thank the respondent for their time. Turn off the recorder.

Interview: Country Evaluation - Sub-county/District Level

(Please complete the Cold Chain Equipment Log Table before this Interview)

Note: Please include all types of equipment (CCE, RTMD, Cold boxes) being procured through CCEOP.

Introduction

1. Could you please describe to me your role within CCEOP? In In case you are not aware, CCEOP is the mechanism through which NVIP/MOH is procuring and distributing cold chain equipment across Kenya with Gavi assistance over the next 2 years.

Existing CCE and Maintenance (Baseline)

We would first like to talk about the existing CCE in your sub-county.

2. In the past 3 months, how often did you hear about CCE problems at health facilities in this sub-county? Can you tell us about some of these problems? [*Probe: If there were problems, how were those problems resolved?]*
3. How is CCE generally maintained? Is there a maintenance plan? [Probe: What is the role of health staff in maintenance? Are there trained technicians? Do they travel to the facilities or does the equipment come to the sub-county level? How are transport and spare parts provided?]
4. What are some of the challenges with maintaining cold chain equipment?
5. To what extent does the cold chain equipment impact vaccine availability in health facilities in this sub-county? [*Probe for examples]*
6. What about the impact on immunization coverage? *[Probe for examples]*
7. And the impact on vaccine wastage rate? *[Probe for examples]*

Operational Deployment Plan

We would now like to get some information on the new CCE to be installed in your sub-county.

8. In what way were you involved in selecting and prioritizing facilities in your sub-county? If so, what methods were used to select the prioritized facilities? [Probe: *How do these facilities and location sites relate to coverage and equity?*]
9. How were you involved in the selection of the equipment?

10. What information have you received on the distribution of new CCE in your sub-county? Please provide details of the timeline and distribution plan and your involvement in the distribution.

11. Describe how you plan to coordinate with the installation team. How have you been engaged with them so far? [Probe: Who is responsible for what actions and the resources available for it?]

Maintenance of CCE

Now I would like to discuss the maintenance of the equipment to be distributed.

12. Can you tell us about plans for maintenance and repairs of the new equipment? [Probe: Who is responsible for what actions and what are the resources available for it?]
    [Probe for the role of service bundle providers/distribution agents?]

Supervision visits and training

13. What plans do you have for monitoring the installation and performance of the newly installed CCE? [*Probe for equipment, stock management practices, immunization service provision].*

14. Who will be trained in operation and maintenance? What do you know about the process?

15. How is training being planned for health workers on preventive maintenance? [Probe for use of manuals and training to use them]

Satisfaction

16. Overall, how satisfied are you with the choice of the new CCE? Can you explain why?

17. What, if anything, would you change about the planning and proposed implementation process? [*Probe*: *What is working well? What could be done differently?*]

18. Is there anything else you want to discuss?

Thank the respondent for their time. Turn off the recorder.

Cold Chain Equipment Log

This table is to be completed prior to the Sub-county Depot and Health Facility Interviews.

Note: Please include all types of equipment (CCE, RTMD, Cold boxes) being procured through CCEOP for this depot/facility that you will receive.

**Equipment**	**Brand and Model**	**Date of Installation/ Acquisition**	**Status** Functional/Non-functional
Existing Cold Chain Equipment, RTM, cold boxes [Note which CCE have RTM/Fridge-tag]			
Anticipated Cold Chain Equipment (in the next 12 months) PLEASE ADD LIST OF EQUIPMENT BASED ON COUNTRY DEPLOYMENT PLAN IN COLUMN 1 BEFORE THE INTERVIEW			

Interview: Country Evaluation - Health Facilities

(Please complete the Cold Chain Equipment Log Table before this Interview)

Note: Please include all types of equipment (CCE, RTMD, Cold boxes) being procured through CCEOP for this facility that you will receive.

Introduction

1. Could you please describe to me your role within CCEOP? In case you are not aware, CCEOP is the mechanism through which NVIP/MOH is procuring and distributing cold chain equipment across Kenya with Gavi assistance over the next 2 years.

Existing CCE and Maintenance (Baseline)

We would first like to talk about the existing CCE in your health facility.

2. In the past 3 months, how often did you hear about CCE problems at this health facility? Can you tell us about some of these problems? [*Probe: If there were problems, how were those problems resolved?]*
3. How is CCE generally maintained? Is there a maintenance plan? [*Probe: What is the role of health staff in maintenance? Are there trained technicians? Do they travel to the facilities or does the equipment come to the sub-county level? How are transport and spare parts provided?*]
4. Do you have a maintenance log book for the CCE? [*Note to interviewer: Check for any maintenance records and if they have been recently used. If no book, what are the factors preventing you from using a log book?*]
5. What are some of the challenges with maintaining cold chain equipment?
6. To what extent does the cold chain equipment impact vaccine availability in health facilities in this sub-county? [*Probe for examples]*
7. What about the impact on immunization coverage? *[Probe for examples]*
8. And the impact on vaccine wastage rate? *[Probe for examples]*

CCE Deployment

We would now like to discuss the new CCE installed through CCEOP.

9. Tell us about the information you have on the new CCE you will be receiving. [*Probe for source of information]*. Did that information change over time? If so, when and why?

10. What information have you received on the distribution of new CCE to this facility? Please provide details of the timeline and distribution plan and your involvement in the distribution.

11. How were you involved or consulted when your health facility was identified for new CCE?

12. Describe how you plan to coordinate with the CCE installation team. How have you engaged with them so far? [*Probe: Who is responsible for what actions and the resources available for it?]*

13. What type of training or orientation do you think is required to operate and maintain the new equipment?

CCE Maintenance Plan

14. Can you tell us about plans for maintenance and repairs of the new equipment? [Probe: Who is responsible for what actions and what are the resources available for it?][Probe for the role of service bundle providers/distribution agents?]

Training and supervision

15. How do you plan to monitor the installation of the new CCE? What about monitoring the performance of this equipment? [*Probe for equipment, stock management practices, immunization service provision]*.

16. What do you know about the process for training on operation and maintenance?

17. How is training being planned for preventive maintenance? [Probe for use of manuals and training to use them]

Satisfaction

18. Overall, how satisfied are you with the choice of the new CCE? Can you explain why?

19. What, if anything, would you change about the planning and proposed implementation process? [*Probe*: *What is working well? What could be done differently?*]

20. Is there anything else you want to discuss?

Thank the respondent for their time. Turn off the recorder.
