# Supplementary material for: Ensuring vaccine potency and availability: how evidence shaped Gavi's Immunization Supply Chain Strategy
Source: BMC Health Serv Res. 2022 Oct 7;22:1237. doi: 10.1186/s12913-022-08616-9 (PMC9540167; doi:10.1186/s12913-022-08616-9)
Supplement: Supplementary file 2 — Additional file 2. [file 12913_2022_8616_MOESM2_ESM.pdf]

JSI RESEARCH & TRAINING INSTITUTE, INC.

Evaluation of the Cold Chain Equipment Optimization Platform (CCEOP) - Endline

Key Informant Interview Guide

November 10, 2020

Submitted by the CCEOP Evaluation Team to Gavi, the Vaccine Alliance

JaRco Consulting

Research And Development Solutions

SVI STAT VIEW International

Table of Contents

Informed Consent Form for Respondents 3
Interview: Country Evaluation - UNICEF Country and Other Country Partners including PMT members 5
Interview: Country Evaluation – National level 10
Interview: Country Evaluation – National level/Finance (NEW) 15
Interview: Country Evaluation –County Level 16
Interview: Regional Store (NEW) 21
Interview: Country Evaluation – Sub-county Level 22
Interview: Country Evaluation - Health Facilities 27
Interview: Country Evaluation - Service Bundle Providers 32

Informed Consent Form for Respondents

Part 1: Information Sheet

Investigator's statement: JSI, along with our country-level implementation partner, Jarco, has been commissioned by Gavi and has been conducting an independent evaluation to assess the relevance, effectiveness, efficiency, outcomes, and sustainability of its Cold Chain Equipment Optimisation Platform (CCEOP) in selected countries since 2018.

This evaluation will assess the progress of the CCEOP against its original objectives. Results will be used to help Gavi improve the design of the Platform, including Gavi's cold chain equipment market-shaping strategies, and support the three countries (Guinea, Kenya, and Pakistan), as well as other Gavi countries implementing the Platform.

We are interviewing people at the global and country level for the endline evaluation to fully capture the outcomes and effect of CCEOP planning, procurement, and implementation activities. Please provide us your honest opinion as both positive and negative perceptions are equally valuable.

What happens in this interview?

As part of this study, you will participate in an in-depth interview that will take about 45-60 minutes. The data collection team will audio-record all responses to questions and take detailed notes. Your name or personal information will not be linked to the data collected or identified in any way. Only aggregate rather than individual data will be shared in reports.

What are the benefits of participating in this interview?

There is no direct benefit, including financial benefits, to you for participating in this interview. However, the answers provided will ultimately help to improve and sustain the coverage and equity of immunization programs in countries implementing the Platform.

Who should I call if I have questions? [PLEASE ADD LOCAL CONTACT INFO]

Please contact the following person for any additional information on this study:
Soumya Alva
Project Lead, CCEOP Evaluation
2733 Crystal Drive
4th Floor
Arlington, VA 22202, USA
Tel: +1 703 528-7474
Email: Soumya_alva@jsi.com

Can I refuse to be part of the study?

Participation in the study is completely voluntary. You can say no now, or leave the study at any time later. You can also refuse to answer any question asked.
Do you have any questions? Do you consent to participate in this interview? Can this interview be recorded?

Part 2: Certificate of consent

Interviewer's statement: I have accurately read aloud the information sheet to the potential respondent, and to the best of my ability made sure that the subject understands the procedures. I confirm that the subject was given an opportunity to ask questions which were answered correctly and to the best of my ability. I confirm that the individual was not coerced into giving consent, and the consent has been given freely and voluntarily.

**Date:**	____________________
**Name of Interviewer obtaining verbal consent:**	____________________
**Signature of Interviewer obtaining verbal consent:**	____________________
**Name of Respondent (to be written by the interviewer):**	____________________
**Position of Respondent:**	____________________
**Length of time in this position:**	____________________

Interview: Country Evaluation - UNICEF Country and Other Country Partners including PMT members

[Note: Questions below specify "last two years". Update this time period in all questions based on country deployment schedule to reflect when new equipment was installed].

Introduction

1. Can you please describe to me your role in CCEOP? Are you a member of the Project Management Team (PMT) and what is your organization's role? [*Probe: What activities were you involved in? How active has the PMT been since last deployment?]*

Engagement with Gavi and other Alliance partners

We would like to discuss the engagement of the UNICEF Country and Regional offices/CHAI with Gavi and other Alliance partners with regard to CCEOP and to the upcoming deployment Kenya.

2. How often did you communicate with Gavi and the Alliance partners throughout the CCEOP planning process? What were the reasons for this communication? How effective was the communication across the planning phase?
3. What mechanisms, if any, were set up to communicate feedback on equipment performance and satisfaction levels over time? How were these mechanisms used? How could these mechanisms be improved?
4. Regarding the suspended Vestfrost CCE models: What communications did you receive about CCE models suspended by the WHO Performance, Quality, and Safety (PQS) team in 2019? What have you seen of the corrective action plan?
5. Based on your experience with communication during the last deployment, what could be done to improve communication for future deployments?

Service Bundle Providers

6. What is your opinion of the bundled services provided by the SBPs? And of the quality standards of the services provided? [*Probe: What enabling factors have contributed to successes of the bundled service? What barriers have been observed related to the bundled service?*]

7. How do you feel about the delinking of service bundle providers that is being discussed for the next phase of CCEOP? What could be some of the advantages of delinking? What are some of the challenges that may come up?

Upcoming Deployment Planning

We would like your views on the country application process for the upcoming deployment including selection of equipment. [Note: Please include all types of equipment (CCE, RTMD, Cold boxes) being procured through CCEOP.

8. In the most recent application, what were the stated gaps for improved cold chain equipment? How did the application address those gaps?
9. What were the key criteria for selecting CCE manufacturers and models for the upcoming deployment? What inputs were used for these decisions of equipment type, brand and model? What were the main sources of information? [*Probe for specific tools/guides.*] Who made these decisions?

    a. How were these considerations different from the earlier rounds of CCEOP equipment procurement? [*Probe: Why were those considerations made? Probe if cost was a consideration. If yes, was total costs of ownership (TCO) considered or purchase price?]*

    b. What was the timing of these changes (if any)? *[Note: try to get an overview or timeline of changes made over the planning period, with dates.]*

    c. How did your experience with equipment procured in previous years influence this decision?

    d. How has CCEOP contributed to the change in CCE selection (either negatively or positively)?

10. What types of equipment were chosen for years 2/3? If the equipment type or manufacturer is different from year 1, what were the primary reasons for changes? What information did you not have that you would have liked during equipment selection? [*Probe: Why were those considerations made?]*
11. How did the most recent application differ from the original proposal? How and why were these changes made? What changes do you anticipate needing to be made to future applications, given the experiences to date?
12. How did you prioritize counties, sub-counties and health facilities for new CCE for the upcoming deployment? What were the criteria? [*Probe: How did the most recent rapid CCE inventory influence the way you made equipment choices in your CCEOP application?]*

13. How will equipment delivery, installation, preventive maintenance and repairs take place for the equipment arriving for years 2/3? [*Probe: Who will be responsible for each activity? How will the activities be monitored? For maintenance, who will provide spare parts? Who will pay for them]*

14. What specific communication have you received regarding equipment warranties? Is it clear what is and is not covered?

Outcomes/Results

Please answer the following questions about any changes that took place in the last two years [Note: Update this time period in all questions based on country deployment schedule to reflect when new equipment was installed].

15. In a broad sense, how has the COVID-19 pandemic impacted the immunization program? *[Probe: HR capacity, session frequency, vaccine availability]*

16. Has the COVID-19 pandemic affected CCEOP planning and deployment? If so, in what way?

17. Over the past two years how has the immunization supply chain changed in Kenya at the national, regional, county and lower levels? [*Probe about any changes in service provision, forecasting, stocks, staffing, data recording, training, etc.*] If any changes, has CCEOP contributed to this change/these changes, either negatively or positively? If so, how? What about any other factors?

18. What has been the effect of the COVID-19 pandemic on the immunization supply chain? How has it affected vaccine stock availability? Have there been any new approaches introduced to lessen COVID disruptions to supply chain functioning and CCE?

19. How has national-level management of the cold chain changed over the past two years? Has CCEOP contributed to this change, either negatively or positively? If so, how? *[Probe: in regards to corrective and preventive maintenance, more cold chain space available.]*

20. Besides cold chain equipment, has CCEOP had an effect on any other aspects of the supply chain, such as data collection and use, maintenance, the design of the supply chain system, or human resource aspects? [*Probe: How has maintenance changed, if at all? Has there been any change in temperature monitoring or tracking distribution?*]

21. What have been unintended (positive and negative) consequences of the CCEOP for Kenya [*Probe: Old, obsolete equipment remaining in facilities, less utilization of ministry engineers, etc.]*?

Sustainability

22. How has CCEOP impacted the country's immunization program? In what way, if any, has the new CCE contributed to a financially viable and sustainable system? To what extent have procedures been established for the successful running of the program? *[Probe for planning procedures, ongoing management of the program, development of institutional systems such as the logistics working group.]*

23. In your opinion, has CCEOP had any effect on the functioning of the Logistics Working Group? [*Probe: in terms of meeting frequency, responsibilities or meeting content*]

24. What is the long-term plan for CCE requirements in the country? *[Probe: Has the inventory been updated? Has the gap analysis plan been updated? Is the country looking at the placement of CCE for better access to communities?]*

25. Is there a long-term plan for new vaccine introduction? If yes, can you describe it to me?

26. What impact will the introduction of new vaccines have on CCE capacity? [*Probe: Do you think the new CCE are sufficient to accommodate the introduction of new vaccines? Why or why not?]*

27. What are long term plans for maintenance and repair of CCE after the warranty period? *[Probe: What are plans for refresher training for long term repair and maintenance? Where are spare parts managed and who is responsible for procuring them?]*

28. How much influence has CCEOP had on EPI policies including considerations for the next comprehensive multi-year plan?

29. How have past cost savings from selected CCE contributed to other activities in the immunization supply chain? What are the plans for future savings from CCEOP?

30. In your opinion, how best can funds for joint investment continue to be used towards CCE as part of cold chain management?

Satisfaction

31. On a scale of 1 to 5, 1 being not at all satisfied and 5 being completely satisfied, what is your level of satisfaction currently with the equipment you have received and its current level of functioning? Can you give us some reasons for your response?

32. What changes do you see now with the arrival of the new CCE compared to the CCE the country had previously?

33. How would you rate the level of efficiency of the CCEOP implementation process in terms of cost and time? [*Probe specifically for deployment, installation, corrective maintenance.]*

34. What, if any, contextual factors (outside the program, such as environmental, political, human resources-related, etc.) do you think may affect CCEOP in achieving its objectives in this country?

35. What changes to CCEOP would you recommend to ensure greater satisfaction overall? [*Probe for procurement, deployment, maintenance, etc*.]

36. Is there anything else you want to discuss?

Thank the respondent for their time. Turn off the recorder.

Interview: Country Evaluation – National level

Note: Collect HMIS data, possibly from timepoints corresponding to baseline, midline and endline to look at trends

Introduction

1. "Describe their past role"

(Example: In the past you have been in the position of XX and have been a PMT member to facilitate the coordination of the planning and implementation of CCEOP, during procurement, CCE delivery and installation). Tell me about your involvement during the first round of deployment and now during the preparation of the next round of deployment.

Cold chain equipment *[Probe for equipment acquired through CCEOP and other sources including older equipment. Additionally if most significant change (MSC) responses are related to COVID-19 disruptions, add probes about changes before March 2020]*

2. Looking back at the last 2 years, what is the most significant change you have seen with the introduction of the new CCE in terms of how well they are performing? [*Probe: In terms of functioning or not, need for preventive or corrective maintenance, fixing any problems with the CCE, temperature stability, etc.- benefits noticed in terms of cost savings*]
3. What is the most significant change you have seen in the vaccine supply chain system in the last two years? *[Probe: preventive and corrective maintenance, inventory, temperature tracking]* Why is this significant to you?
4. Over the last two years, what has been the most significant change you have seen in immunization service delivery? What do you think are the contributing factors to this change?
5. In your opinion, what is the most significant change in temperature monitoring in the last two years? [*Probe: type of devices used for temperature monitoring, if remote or FridgeTag, any behavior practices changed, etc.]*
    a. In your opinion, what are the contributing factors to this change in temperature monitoring?

Maintenance ***[Probe for equipment acquired through CCEOP and other sources including older equipment]***

6. From your point view, what has been the most significant change in preventive and corrective maintenance levels and procedures in the country for new and existing equipment in the last two years?
    a. What do you think are the contributing factors to this change?
7. In the past two years, what is the most significant change you have noticed related to maintenance technicians' training and performance? Why is this change significant to you?
8. In your opinion, what is the most significant change in maintenance you have noticed with warranties over the last two years?
    a. Why is this change significant and does it correspond to your expectations of what was covered under the warranty?
9. In your opinion, have there been any changes in the maintenance of existing CCE (non CCEOP) because of the changes in maintenance of the new CCE? Please describe these changes.
10. Kenya received some of the suspended Vestfrost CCE models: What communications did you receive about CCE models suspended by the WHO Performance, Quality, and Safety (PQS) team in 2019? What have you seen of the corrective action plan?]

Service bundle providers

11. Since installation, what is the most significant change have you seen over time in the SBPs' service provision? [probe on enabling factors or barriers]
12. How do you feel about the delinking of service bundle providers for the next phase of CCEOP? What are some of the advantages of delinking? What are some of the challenges that may come up?
13. What are the plans for preventive and corrective maintenance after the SBP warranty period ends? *[Probe existence of a long-term plan, is there financing secured for the plan?]*

Vaccine Supply Chain Management (inventory management)

14. In your opinion, in the last two years, what has been the most significant change in vaccine stock availability in facilities in Kenya?

15. In the last two years, what has been the most significant change in vaccine supply chain management? *[Probe for vaccine forecasting, procurement, inventory management, distribution, vaccine wastage]* Has there been any significant change in vaccine stock availability? In your view, what are the contributing factors? *[Probe for changes in the last 6-9 months (COVID-specific) as well]*

16. What has been the effect of the COVID-19 pandemic on the immunization supply chain? How has it affected vaccine stock availability? Have there been any new approaches introduced to lessen COVID disruptions to supply chain functioning and CCE?

Other Outcomes

17. In your opinion, what was the most significant change in the immunization program since the COVID-19 pandemic? *[Probe: HR capacity, session frequency, vaccine availability]*

18. What was the most significant change in CCEOP planning and deployment since the COVID pandemic? Why is this significant to you?

19. How has CCEOP complemented other investments in immunization in the country? *[Probe: from other development partners past, current and planned investments]*

20. What have been unintended (positive and negative) consequences of the CCEOP for Kenya? *[Probe: Old, obsolete equipment remaining in facilities, less utilization of ministry engineers, etc.]*

Second Deployment

21. What was the most significant change from the first round of CCEOP planning and deployment? *[Probe role of PMT, ODP, maintenance plan, training plan]*

22. At baseline, the process of selecting equipment and understanding the pricing for each piece of equipment was challenging during the first phase of planning. What in your opinion, did you find challenging this time?

    a. In your view, what was the most significant change in facility and equipment selection for this round? Why is this change significant to you?What were the 3 main factors that contributed to this change?

23. What was the most significant change in information availability to advise the second proposal compared to the first proposal? *[Probe: including information used for the ODP and deviation plan]*

Sustainability

24. [For example in the Guinea midline this was reported the tool for tracking inventory. Other countries please note if there are examples of other tools] Has the WHO inventory tool been continuously updated to inform the long-term plan for CCE requirements in the country? *[Probe: Has the gap analysis plan been updated? Is the country looking at the placement of CCE for better access to communities?]*

25. In 2019, there were plans to introduce the HPV vaccine as well as conduct malaria vaccine trials]. In your opinion, what is the most significant change in storage capacity and distribution due to the introduction of these vaccines? What is the long term plan for additional vaccine introduction?

[To understand whether there is an incentive for choosing a more cost-effective CCE and understand the use of cost savings?]

26. How have past cost savings from procuring selected CCE contributed to other activities in the immunization supply chain? What are the plans for future savings from CCEOP?

27. How much influence has CCEOP had on EPI policies including considerations for the next comprehensive multi-year plan?

28. In your opinion, how best can funds for joint investment continue to be used towards CCE as part of cold chain management?

29. What, if any, contextual factors (e.g those outside the EPI program such as environmental, political, human resources-related, etc.) do you think may affect CCEOP in achieving its objectives?

30. In your opinion, what has been the most significant change in the role played by Logistics Working Group? *[Probe: in terms of meeting frequency, responsibilities or meeting content]*

Satisfaction

31. What type of support did you receive from the various stakeholders in the implementation of CCEOP post implementation of the 1st phase, in preparing the second proposal and in planning the next round of deployment? *[Probe about support from UNICEF/Supply Division, UNICEF country office and the PMT, partners]*

    a. Are you satisfied with the support from each of these stakeholders? *[Probe: Please explain why]*

32. On a scale of 1 to 5, 1 being not at all satisfied and 5 being completely satisfied, overall, what is your level of satisfaction currently with the equipment you have received? Please explain your reasons.

33. How would you rate the level of efficiency of the CCEOP implementation process in terms of cost and time?

34. What, if anything, would you change about the implementation of CCEOP to improve satisfaction overall? *[Probe: What worked well? What did not work well? What are some lessons learnt? What could be done differently? How will these lessons learnt inform the next phase of CCEOP?]*

35. What are future plans for the next phase of CCEOP? When will equipment selection take place? How will facilities be selected? When will the equipment be ordered and when will it be deployed? (i.e. get a timeline of events).

36. Have you received any information from Gavi about possible changes coming to CCEOP that may have implications for financing (i.e. how money will be set aside for CCE and expectations for joint investment?) If yes, please explain.

37. Is there anything else you want to discuss?

Thank the respondent for their time. Turn off the recorder.

Interview: Country Evaluation – National level/Finance (NEW)

The purpose of this interview is to understand to what extent joint investment for CCEOP contributes to sustainability and ownership.

1. Where are financial decisions for the joint investment made? Who makes them?
2. Are there any general revenues at the central level (i.e. national resources) used towards the joint investment for the procurement of CCE in the country?
3. (If financing and management responsibilities are decentralized in the health system) How has decentralization impacted the use of general revenues for immunization services? Has decentralization had any effect on the joint investment for CCE?
4. How do the characteristics of co-financing for vaccines and joint investment for CCE impact sustainability of vaccines and CCE in the country?
5. In your view, to what extent were discussions and decisions on joint investment led by the Ministry of health/country?
6. In your view, what is the most significant change you have seen with the joint investment for CCE?
    a. Why is this significant to you?
7. In your opinion, how best can funds for joint investment continue to be used towards CCE as part of cold chain management?

Thank the respondent for their time. Turn off the recorder.

Interview: Country Evaluation –County Level

Introduction

1. Can you please tell me your name, position title and tell me how long you have been in your current position at the county?

2. Can you please describe to me your role throughout the procurement of CCE, the implementation of the CCEOP, post-deployment and now preparation for the next round of deployment? [*Probe: tracking shipment, shipment clearance, installation of CCE, oversight*]

Immunization Services

3. Tell me about the immunization services you offer in facilities in this county. Are there any improvements and/or changes over the last two years? If yes, what improvements and/or changes have you observed? *[Probe: access, coverage, populations reached/not reached]*

4. What are some of the challenges you have faced over the last two years? What are your current challenges?

5. Have you observed any changes in immunization coverage in your county over the last two years? If yes for which antigens? *[Ask for any reports/data]*

6. Have you observed any changes in drop out rates in your county over the last two years? *[Ask for any reports/data]*

ODP implementation

We would like to talk about the most recent CCEOP deployment.

7. To what extent has any of the new equipment that was installed in the last round of deployment (2018) been moved since it was originally installed? If so, what was the reason for moving it? What about old equipment?

8. How are you monitoring the performance of the new CCE? *[Probe for equipment, maintenance, stock management practices, immunization service provision, temperature monitoring].*

Cold chain equipment *[Probe for equipment acquired through CCEOP and other sources]*

9. What changes have you seen in the performance of the new CCE compared to the CCE it replaced? *[Probe: In terms of functioning or not, need for preventive or corrective maintenance, fixing any problems with the CCE, temperature stability, etc.]*

10. How has the new cold chain equipment affected facilities and the sub-county depots in your county? What changes have you seen? [*Probe for examples.]*

11. Have there been any other savings or benefits in terms of running costs through replacement of obsolete equipment? *[Probe: savings on gas or electricity, maintenance]*

12. To what extent has the CCEOP contributed to expanding CCE at the county/provincial and health facility level, such as being able to add or upgrade more facilities? How many have been able to be added or upgraded through CCEOP support? How has extension of coverage areas changed if at all?

13. In what ways, has the new/upgraded CCE affected/changed service delivery at facilities? Has it changed the way outreach services were previously provided?

Maintenance *[Probe for equipment acquired through CCEOP and other sources including older equipment]*

14. How much corrective maintenance has the new equipment needed since installation (in 2018)? And what have you done for preventive maintenance?

    a. If corrective maintenance has been required, how has the equipment warranty factored into the maintenance? [*Probe: Did the warranty cover some or all of the repairs? Why or why not? What was covered? What was not covered? Explain the process.*]

15. If there is a problem with an older piece of CCE, what is the protocol for repair? *[Probe for what the process for repair is, and who is scheduled to repair]*

16. How has the new CCE affected maintenance requirements for county and sub-county maintenance teams? *[Probe: How have the operational costs (maintenance and repair, gas/propane refill requirements) changed since installation of new equipment?]*

17. What is your understanding of the length of the warranties for the various new CCE and of what the equipment warranties include? *[Probe for warranty duration in years by equipment type and manufacturer – i.e. SDD vs. AC.]*

18. What is the process by which corrective maintenance of CCE is conducted, for example when something needs to be fixed? Is there a maintenance plan, and if so at what level (national/sub-national)? Is maintenance timely? How are spare parts managed? What support is provided for corrective maintenance? *[Probe for new and old equipment] [Probe for the person who maintains the spare parts, where are they kept, how do they get to facilities where they are needed, how to they find out about what equipment needs maintenance].*

19. How are technicians equipped to operate and maintain the new equipment? Have they received any training in the last year?

20. What is your opinion of the system for maintenance and repair for CCE? What works well? What are some of the challenges? What would improve the system?

21. How are staff in the health facility equipped to operate the CCE and provide basic preventive maintenance? What is the training process on the new equipment for users to ensure correct maintenance practices? *[Probe for use of manuals/SOPs and training to use them]*

22. What type of supervision and feedback do staff receive on preventive maintenance?

23. How has preventive maintenance changed since the installation of the new equipment? *[Probe: preventive maintenance for solar panels]*

24. If there has been any breakdown with the new CCE, what were some of the main reasons for equipment breakdown in CCEOP supported sub-county/districts and facilities? How was the breakdown reported and what was the process for repair?

25. If there was any breakdown with older CCE, what were some of the main reasons for equipment breakdown? How was the breakdown reported and what was the process for repair?

26. What specific communication have you received regarding equipment warranties? Is it clear what is and is not covered?

27. What are long term plans for maintenance and repair of CCE after the warranty period? *[Probe: What are plans for refresher training for long term repair and maintenance? What materials have been developed? Where are spare parts managed and who is responsible for procuring them?]*

Other Outcomes

28. In a broad sense, how has the COVID-19 pandemic impacted the immunization program? *[Probe: HR capacity, session frequency, vaccine availability]*

29. Has the COVID-19 pandemic affected CCEOP planning and deployment? If so, in what way?

30. In the last two years, have there been any changes in vaccine stock availability in facilities in this county? If yes, please explain. Has the new CCE impacted stock availability? Please explain why or why not.

31. In the last two years, have there been any change in vaccine wastage in facilities in this county? If yes, please explain. What about other stock management and reporting procedures?

32. How has the introduction of [HB=41, Mars=25, Kitui=52]number of CCE impacted distribution of vaccines in this county?

33. What has been the effect of the COVID-19 pandemic on the immunization supply chain? How has it affected vaccine stock availability? Have there been any new approaches introduced to lessen COVID disruptions to supply chain functioning and CCE?

34. How do you monitor the temperature of equipment in this county (RTMD or FridgeTag)? If you do have RTMDs, do you have access to the temperature data generated by those devices? If so, how do you use it? How do you access data from FridgeTag? And how do you use it?

35. What happens when the temperature in a piece of CCE gets too hot or cold either at the sub-county depot or in a facility? Does anyone receive an SMS alert from the RTM? Please explain the steps taken and give examples from your experience.

36. What has been the most significant change related to CCE and vaccination in the last two years? Could you tell me more about that? What has been the effect? Please provide specific examples.

Satisfaction

37. Have you received support from the national level (NVIP) in implementation of CCEOP (implementation of new cold chain equipment)? If so, please describe. What about from others such as UNICEF, other partners or the PMT (i.e. CHAI, WHO etc.)? Are you satisfied with the support received from them? *[Probe: Please explain why/why not]*

38. On a scale of 1 to 5, 1 being not at all satisfied and 5 being completely satisfied, overall, what is your level of satisfaction currently with the equipment you have received? Please explain why.

39. What, if anything, would you change about the ODP implementation or CCE received? *[Probe: What worked well? What could have been done differently? What are some lessons learnt?]*

40. What are future plans for the next phase of acquiring equipment through CCEOP? [*Probe for knowledge of delinking of SBP and the implications.]*

    a. In what ways do you think the delinking of SBPs will affect your county ?

41. Compare and contrast the use of service bundle providers with use of government biomedical engineers and cold chain technicians for corrective maintenance during the equipment warranty period. What are some of the strengths of the service bundle providers? What are some of the weaknesses?

Second Deployment Planning

42. What role have you played in the planning process for the next round of CCEOP? *[Probe: involvement with the cold chain inventory, development of the ODP, or other planning activities]*

43. For the next deployment, how were health facilities in your county prioritized for new CCE? What was your role in the process of health facility selection in your county? What role, if any, did you have in the selection of equipment?

Sustainability

44. What is the long-term plan for CCE requirements in the country? *[Probe: has the inventory been updated? Has the gap analysis plan been updated? Is the country looking at the placement of CCE for better access to communities?]*

45. Is there a long-term plan for new vaccine introduction? If yes, can you describe it to me?

46. What impact will the introduction of new vaccines have on CCE capacity? *[Probe: Do you think the new CCE are sufficient to accommodate the introduction of new vaccines? Why or why not?]*

47. Does the county have a plan in place for removal of obsolete equipment? If so, please explain. *[Probe: Is old equipment being disposed or removed? How?]*

48. Is there anything else you want to discuss?

Thank the respondent for their time. Turn off the recorder.

Interview: Regional Store (NEW)

Effects of COVID-19

1. Supply chain and stocks:
   a. How has COVID-19 affected the immunization supply chains (or CCE)?

   b. Have there been any vaccine stock-outs related to COVID-19 or otherwise? If yes, can you explain what happened?
      - ***Probe:*** How has this changed over time (Mar/Apr to May/June to now)?

   c. Have there been any new approaches introduced to lessen COVID disruptions to supply chain functioning and CCE? If yes, what?

Stock Management

2. How do you get vaccine stock to the sub-county store from the regional store (i.e. delivered, fetched, etc.?) How often do sub-county stores get new stock? How has this changed since the arrival of the new cold chain equipment in the sub-county stores?

3. How do you receive requests from sub-county stores for vaccine stock? How do you fill these requests? Describe the process for fulfilling these requests. What is your opinion of the process? Are sub-county stores following procedures? Do they receive training, supervision or feedback on stock management?

4. Have there been stock-outs of any vaccines in the two years? What was the reason for the stock-out? How long did it last?

Interview: Country Evaluation – Sub-county Level

Note: if interviewing the same person as interviewed at baseline or midline, please explain that we will be asking about the time that has elapsed since installation of Gavi/CCEOP equipment.

Introduction

1. Can you please tell me your name, position title and tell me how long you have been in your current position at the sub-county?

2. Can you please describe to me your role in the implementation of the CCEOP (i.e. the implementation of new CCE funded in part by Gavi and the Government of Kenya? [*Probe: installation of CCE, training on maintenance, oversight, development of ODP*]

ODP implementation

3. In a broad sense, how has the COVID-19 pandemic impacted the immunization program? *[Probe: HR capacity, session frequency, vaccine availability]*

4. Has the COVID-19 pandemic affected CCEOP planning and deployment? If so, in what way?

5. What has been the effect of the COVID-19 pandemic on the immunization supply chain? How has it affected vaccine stock availability? In your view, what are the contributing factors? *[Probe for changes in the last 6-9 months]*

We would like to talk about the CCEOP implementation process

6. In what ways have you coordinated with service bundle providers, and the national, county level staff since the new CCE was installed?

7. In the last year, have technicians or health facility staff received any additional training on preventive or corrective maintenance for the new CCE? If so, please describe.

8. How are you monitoring the performance of the new CCE, its maintenance, stock management practices and/or immunization service provision?

9. To what extent has the equipment remained in the facilities where it was allocated in the ODP? Please provide examples and explanation for equipment that has been moved.

Cold chain equipment *[Probe for equipment acquired through CCEOP and other sources. If unfamiliar with CCEOP by name, please refer to "new CCE"]*

10. How do you monitor the temperature of equipment in the facilities in this sub-county (FridgeTag or Remote Temperature Monitoring)? Do you have access to the temperature data generated by the remote temperature monitoring devices? If so, how do you use that data? If you use FridgeTag, how do you access the data? And how do you use that data?

11. What happens when the temperature in a piece of CCE gets too hot or cold either at the sub-county depot or in a facility? Does anyone receive an SMS alert from the RTM? Please explain the steps taken and give examples from your experience.

12. What changes have you seen in the performance of the new CCE compared to the CCE it replaced? *[Probe: In terms of functioning or not, need for preventive maintenance, fixing any problems with the CCE, temperature stability, etc.]*

13. How has the new cold chain equipment affected facilities in the sub-county and the sub-county store? What changes have you seen? *[Probe for examples]*

14. Have there been any other savings or benefits in terms of running costs through replacement of older equipment (e.g. savings on gas or electricity)?

15. To what extent has the CCEOP/new CCE contributed to extension of the supply chain, replacement (i.e. rehabilitation), and expansion of CCE at sub-county and health facility levels? *[Probe: How many more facilities have added and/or upgraded equipment with new CCE? Extension of geographic coverage? Areas previously not reached?]*

16. How has the introduction of X number of CCE impacted distribution of vaccines in this sub-county?

17. How are you tracking progress in immunization service provision now that you have an increased number of facilities providing immunization?

18. How, if at all, has the new CCE contributed to the ability of facilities to conduct outreach to hard-to-reach populations? By outreach, we are referring to immunization activities that take place outside of the normal immunization services offered in the facility. These activities are conducted to reach hard to reach populations or populations that do not come to the facility for immunizations.

19. In what ways, has the upgraded/new CCE affected/changed service delivery at facilities? Has it changed the way outreach services were previously being provided? If so, how? How have mobile outreach sessions (where applicable) been affected?

Stock Management

20. How do you get vaccine stock to the sub-county store from the regional store (i.e. delivered, fetched, etc.?) How often do you get new stock and how much do you usually get? How has this changed since the arrival of the new equipment?

21. How do you receive requests from facilities for vaccine stock? How do you fill these requests? Describe the process for fulfilling these requests. What is your opinion of the process? Are facilities following procedures? Do they receive training, supervision or feedback on stock management?

22. Does the sub-county receive feedback from higher levels on the inventory management practices? If so, please give examples.

23. In the last two years, have there been any changes in vaccine stock availability in facilities in this sub-county or in the sub-county store itself? If yes, please explain. Has the new CCE impacted stock availability in facilities or in the sub-county store? Please explain why or why not.

24. Have there been stock-outs of any vaccines in the last year? What was the reason for the stock-out? How long did it last?

25. What are the normal procedures for vaccine stock management? *[Probe for cards/ledger books used. Probe for explanation of all activities, from request to receipt, daily management and recording]*. How has the new CCE impact storage of vaccines? (Kenya- provision of trays for better organization)

26. What are your observations on the vaccine stock management procedures? What are some of the challenges?

27. What type of feedback or supervision do you receive from higher levels on your stock management procedures, including documentation? Please explain. Have you taken this feedback into account and altered your practices? Please give an example.

28. In the last two years, have there been any changes in vaccine wastage in facilities in this sub-county or in the sub-county store itself? If yes, please explain. What about other stock management and reporting procedures?

Maintenance *[Probe for equipment acquired through CCEOP and other sources including older equipment]*

29. How much corrective maintenance has the new equipment needed since installation? If any breakdown with the new CCE, what have been some of the main reasons for equipment breakdown? What about for existing/older CCE?

1. If corrective maintenance has been required, how has the equipment warranty factored into the maintenance? *[Probe: Did the warranty cover some or all of the repairs? Why or why not? What was covered? What was not covered? Explain the process.]*

30. How has the new CCE affected maintenance requirements for county and sub-county maintenance teams? *[Probe: How have the operational costs (maintenance and repair, gas refill requirements) changed since installation of new equipment?]*

31. What is the process by which corrective maintenance of CCE is conducted, for example when something needs to be fixed? Is there a maintenance plan, and if so at what level (national/sub-national)? Is maintenance timely? How are spare parts managed? What support is provided for corrective maintenance? *[Probe for new and old equipment] [Probe for the person who maintains the spare parts, where are they kept, how do they get to facilities where they are needed, how to they find out about what equipment needs maintenance].*

32. What is your opinion of the system for maintenance and repair for CCE? What works well? What are some of the challenges? What would improve the system?

33. How are technicians equipped to operate and maintain the new equipment? How about existing equipment?

34. What type of supervision and feedback do staff receive on preventive maintenance in the facilities and at the sub-county store?

35. How are staff in the health facility equipped to operate the CCE and provide basic preventive maintenance? What is the training process on the new equipment for users to ensure correct maintenance practices? *[Probe for use of manuals/SOPs and training to use them]*

36. How, if at all, has preventive maintenance of CCE changed since the installation of the new equipment? Please provide examples.

37. What is your understanding of the length of the warranties for the various new CCE and what the equipment warranties include? *[Probe for warranty duration in years by equipment type and manufacturer – i.e. SDD vs. AC.]*

38. If there was a problem with a piece of CCE while under warranty, what is the protocol for repair? *[Probe for what the process for repair is, and who is scheduled to repair]*

39. If there was a problem with an older piece of CCE, what is the protocol for repair?

40. What are long term plans for maintenance and repair of CCE after the warranty period? *[Probe: What are plans for refresher training for long term repair and maintenance?]*

41. Does the sub-county have a plan in place for removal of obsolete equipment? If so, please explain. *[Probe: Is old equipment being disposed of or removed? If so, how and by whom?]*

42. What has been the most significant change related to CCE and vaccination in the last two years? Could you tell me more about that? Why do you think this changed?

Satisfaction

43. How would you describe the support you have received on the whole process of getting new equipment (through CCEOP if they are familiar)? Are you satisfied with the support received? [*Probe for the national, county level? Please explain why/why not*]

44. On a scale from 1 to 5 with 1 being not at all satisfied and 5 being very satisfied, overall, what is your level of satisfaction currently with the equipment you have received? Please explain why. What would you like to change going forward if you had a choice?

45. Is there anything else you would like to discuss?

Thank the respondent for their time. Turn off the recorder.

Interview: Country Evaluation - Health Facilities

Introduction

1. Can you please tell me your name, position and tell me how long you have been in your current position at this facility?

2. Can you please describe to me your role in the management of the new CCE in your facility, from installation to daily maintenance? [*Probe: guiding installation of CCE, preventive maintenance*]

Staffing

3. Can you describe to me the number and type of staff who provide immunization services in this facility? [*Probe: Are these staff paid and full time? Is the staffing sufficient? How has staffing changed over time? Please explain.*]

4. How many staff members have received training in immunization?

Immunization Services

5. Tell me about the immunization services that you provide at this facility. How often are immunizations available? Has immunization service provision changed over time (in the two years)? Which vaccines do you offer? What are some of the challenges you face?

6. In a broad sense, how has the COVID-19 pandemic impacted the immunization program? What has been the effect on the immunization supply chain? *[Probe: HR capacity, session frequency, vaccine availability]*

7. Have there been any new approaches introduced to lessen COVID disruptions to supply chain functioning and CCE?

8. Do you offer outreach services at this facility? Can you describe what a typical outreach session looks like? How often do they occur? What are some of the challenges?

9. Which communities access the outreach sessions? *[Probe: population, distance covered from the facilities]* How have outreach services changed over time?

Cold chain equipment *[Probe for equipment acquired through CCEOP and other sources]*

10. How do you monitor the performance of the new CCE, stock management practices and/or immunization service provision? *[Probe: temperature*

monitoring charts, stock ledgers, monthly reports on stock and immunization coverage]

11. What changes have you seen in the performance of the new CCE compared to the CCE it replaced? [*Probe: In terms of functioning or not, need for preventive maintenance, fixing any problems with the CCE, temperature stability, etc.*]

12. How has the new cold chain equipment affected your facility? What changes have you seen? [*Probe for examples*]

13. Have there been any savings or benefits in terms of running costs through replacement of obsolete equipment? *[Probe: savings on gas or electricity or maintenance]*

14. In what ways has the upgraded equipment affected/changed service delivery at your facility? [*Probe: any changes in frequency of immunization services or number of children immunized?]*

15. How, if at all, has the new CCE contributed to the ability of facilities to conduct outreach to hard-to-reach populations? By outreach, we are referring to immunization activities that take place outside of the normal immunization services offered in the facility. These activities are conducted to reach hard to reach populations or populations that do not come to the facility for immunizations.

Stock Management

16. How do you get vaccine stock in your facility (i.e. delivered, fetched on a moto, etc.?) How often do you get new stock and how much do you usually get? How has this changed since the arrival of the new equipment?

17. In the last two years, have there been any changes in vaccine stock availability in your facility? If yes, please explain.

18. Have there been stock-outs of any vaccines in the last two years? What vaccines? What was the reason for the stock-out? How long did it last?

19. What are the normal procedures for vaccine stock management? *[Probe for cards/ledger books used. Probe for explanation of all activities, from request to receipt, daily management and recording].*

20. Is there a staff member in the facility trained on the standard operating procedures for managing vaccines? How did they receive the training?

21. How do you manage vaccines on a daily basis? [*Probe: How do you prepare them for immunization sessions? Where do you store them during immunization sessions? Where and when do you record what you are using? What do you do with the vials at the end of the day?]*

22. How do you feel about the vaccine stock management procedures? What are some of the challenges?

23. What type of feedback or supervision do you receive from higher levels on your stock management procedures, including on reports you submit? If so, please explain. Have you taken this feedback into account and altered your practices? Please give an example.

24. In the last two years, have there been any changes in vaccine wastage in your facility? If yes, please explain.

Other Outcomes

25. How do you monitor the temperature of equipment in this facility? (If facility has equipment with remote temperature monitoring (RTM) capabilities, ask: Do you have access to the temperature data generated by the RTM? If so, how do you use it? If using FridgeTag, how do you access the data? [*Probe: specifically about historical data and alarms, not just current temperature*] And how do you use the data?

26. What happens when the temperature in a piece of CCE gets too hot or cold? Does anyone from the facility receive SMS alerts from the RTM? Please explain the steps taken and give examples from your experience. *[Probe: for subsequent steps taken?]*

Maintenance *[Probe for equipment acquired through CCEOP and other sources including older equipment]*

27. How much corrective maintenance has the new equipment needed since installation? What type of repairs have been needed?

    a. If corrective maintenance has been required, how has the equipment warranty factored into the maintenance? [*Probe: Did the warranty cover some or all of the repairs? Why or why not? What was covered? What was not covered? Explain the process.*]

28. What is the process by which corrective maintenance of CCE is conducted, for example when something needs to be fixed? Is there a maintenance plan, and if so at what level (national/sub-national)? Is maintenance timely? How are spare parts managed? What support is provided for corrective maintenance? *[Probe for new and old equipment] [Probe for the person who maintains the spare parts, where are they kept, how do they get to facilities where they are needed, how to they find out about what equipment needs maintenance].*

29. What is your opinion of the system for maintenance and repair for CCE? What works well? What are some of the challenges? What would improve the system?

30. How are staff in the health facility equipped to operate the CCE and provide basic preventive maintenance? What is the training process on the new equipment for users to ensure correct maintenance practices? [*Probe for use of manuals/SOPs and training to use them]*

31. Have there been any challenges with the CCE or with the solar panels when conducting preventive maintenance? If yes, what have been these challenges?

32. What type of supervision and feedback do staff receive on preventive maintenance?

33. What is the process by which preventive maintenance of CCE is conducted? What actions are taken? How often and by whom? How it is documented?

34. How, if at all, has preventive maintenance changed since the installation of the new equipment?

35. If there was a problem with a piece of CCE while under warranty, what is the protocol? [*Probe for what the process for repair is, and who is scheduled to repair]*

36. If there was a problem with a piece of older CCE (not under warranty), what is the protocol for repair or corrective maintenance?

37. How has the new CCE changed how you do preventive and corrective management at your facility?

38. Who are the persons who need to be informed if there is need for corrective maintenance or other issues with the CCE? [*Probe for both old and new CCE*]

Satisfaction

39. How would you describe the support you have received on the whole process of getting new equipment? Are you satisfied with the support received? [*Probe for the national, county, sub-county level? Please explain why*]

40. On a scale of 1 to 5, 1 being not at all satisfied and 5 being completely satisfied, overall, what is your level of satisfaction currently with the equipment you have received? Please explain why.

41. On a scale of 1 to 5, 1 being not at all satisfied and 5 being completely satisfied, what is your level of satisfaction with any training provided on preventive maintenance of the new CCE? Please explain.

42. On a scale of 1 to 5, 1 being not at all satisfied and 5 being completely satisfied, what is your level of satisfaction with any corrective maintenance that has been done on the new CCE or corrective maintenance on existing CCE other than CCEOP? Please explain.

43. What has been the most significant change related to CCE and vaccination in the last two years? Could you tell me more about that? Why do you think this changed?

44. Is there anything else you want to discuss?

Thank the respondent for their time. Turn off the recorder.

Interview: Country Evaluation - Service Bundle Providers

Note: *Request any documentation of maintenance activities performed*

Introduction

1. Can you please tell me your name, position and tell me how long you have been in your current position with this company?
2. Can you please describe to me your role in the implementation of CCEOP? [*Probe: delivery of equipment, installation of CCE, training on preventive maintenance, corrective maintenance*]

Now we would like to discuss your role in maintenance of the CCE.

3. Now that the equipment has been delivered and installed, what activities are included as part of your contract for CCEOP? When does your role end?
4. Describe any communication with the PMT since the equipment was installed. [*Probe for frequency and content*]
5. Describe the process of coordination with the manufacturer, UNICEF, and MOH for maintenance of CCE. What about coordination with the lower levels of the health system including health facilities? [*Probe for each level – counties, sub counties and facilities. Was this coordination sufficient and as expected?]*
6. Are there other costs or activities that have come up that were not initially planned for since the equipment has been installed?
7. What is the process to be followed in case there is a maintenance or repair issue with the equipment? What are your responsibilities during the warranty period regarding maintenance?
8. What maintenance activities have you conducted since the equipment was installed? If any breakdown with the new CCE, what have been some of the main reasons for equipment breakdown? (*Ask Vestfrost providers specifically about breakdown of Vestfrost equipment, the specific problem and how it was resolved)*. Were these breakdowns covered by the warranty? Who paid for the repairs?
9. What will be the process for maintenance or repair after the warranty ends?
10. Have there been any joint plans with EPI on how maintenance roles will be transferred once the warranty ends?
11. Have your services after installation included the sustenance of the training you provided (i.e. any plans for refresher training)?

RTM monitoring

12. What is your role in monitoring data captured by the remote temperature monitoring devices? How were these devices set up to send data and to whom? How often is this data reviewed? What is the protocol for sharing the data with the ministry and use of the data by the SBP?

Subsequent Deployments

13. Do you have a role in the upcoming CCEOP equipment deployment? If so, please explain.

Overall Satisfaction and Outcomes

14. During the entire process of CCEOP (from contracting, shipping, installation, training on the equipment, maintenance/repairs), did you face any issues/ challenges? [*Probe for each phase mentioned above*.] How were these resolved?
15. Have there been any positive outcomes or consequences of CCEOP that you did not expect? What were they? Why do you think they happened? (E.g. SBP received more exposure/business due to work with CCEOP, etc.)
16. Have there been any negative outcomes or consequences of CCEOP that you did not expect? What were they? Why do you think they happened?
17. Have you observed any unintended consequences of the new CCE? (Include positive or negative consequences.)
18. Overall, have you been satisfied with the CCEOP process? *[Probe about coordination with ministry, the contracting process, installation, etc.]* Please explain.
19. What would you like to change about CCEOP going forward if you had a choice?
20. Is there anything else you want to discuss?

Thank the respondent for their time. Turn off the recorder.
